# Acid-Sensing Histidine Kinase With a Redox Switch

**DOI:** 10.3389/fmicb.2021.652546

**Published:** 2021-05-20

**Authors:** Shinya Inada, Toshihide Okajima, Ryutaro Utsumi, Yoko Eguchi

**Affiliations:** ^1^Graduate School of Biology-Oriented Science and Technology, Kindai University, Kinokawa, Japan; ^2^The Institute of Scientific and Industrial Research, Osaka University, Ibaraki, Japan

**Keywords:** oxidation, redox, acid, ubiquinone, two-component system, *Escherichia coli*, EvgS, PAS domain

## Abstract

The EvgS/EvgA two-component signal transduction system in *Escherichia coli* is activated under mildly acidic pH conditions. Upon activation, this system induces the expression of a number of genes that confer acid resistance. The EvgS histidine kinase sensor has a large periplasmic domain that is required for perceiving acidic signals. In addition, we have previously proposed that the cytoplasmic linker region of EvgS is also involved in the activation of this sensor. The cytoplasmic linker region resembles a Per-ARNT-Sim (PAS) domain, which is known to act as a molecular sensor that is responsive to chemical and physical stimuli and regulates the activity of diverse effector domains. Our EvgS/EvgA reporter assays revealed that under EvgS-activating mildly acidic pH conditions, EvgS was activated only during aerobic growth conditions, and not during anaerobic growth. Studies using EvgS mutants revealed that C671A and C683A mutations in the cytoplasmic PAS domain activated EvgS even under anaerobic conditions. Furthermore, among the electron carriers of the electron transport chain, ubiquinone was required for EvgS activation. The present study proposes a model of EvgS activation by oxidation and suggests that the cytoplasmic PAS domain serves as an intermediate redox switch for this sensor.

## Introduction

Bacteria utilize two-component signal transduction systems (TCSs) to respond and adapt to fluctuating environmental conditions (Stock et al., 2000). A typical TCS consists of a histidine kinase (HK) sensor and its partner, a response regulator (RR). Input of a specific stimulus to the HK sensor causes autophosphorylation of a conserved histidine residue in the catalytic core. This phosphate group is subsequently transferred to the partner RR; the phosphorylated RR functions mainly by regulating gene expression, which is served as the output.

Most HK sensors are membrane proteins that are localized in the cytoplasmic membrane (Cock and Whitworth, 2007). In a prototypical HK sensor, the extracellular sensor region perceives environmental stimuli and transduces the information via the transmembrane segment to the cytoplasmic region, where the catalytic core resides. Additional protein domains such as HAMP (HK, adenylyl cyclases, methyl-accepting proteins, and other prokaryotic signaling proteins) and PAS (Per-ARNT-Sim) are often found between the transmembrane segment and the catalytic core. These domains transmit the N-terminal signal to the catalytic core and/or perceive additional signal inputs (Zschiedrich et al., 2016). Although a typical HK perceives the stimuli at its extracellular sensor domain, some HK sensors lack the extracellular region (sensors consisting of only transmembrane and cytoplasmic regions), while other HK sensors are cytoplasmic proteins. Thus, in addition to the extracellular sensing domain, signals are also perceived at the transmembrane segment and/or cytoplasmic sensing domains. The specific signals that stimulate HK sensors are unknown in many cases. Far less known are the domains through which the signals are perceived. Even when the signal of an HK sensor is well defined, upon close examination, a different signal approaching another domain may be discovered. Such new information may add to our interpretation of how TCS functions.

The present study aimed to understand the activation mechanism of the acid-responding EvgS HK sensor in *Escherichia coli*. EvgS, together with its cognate EvgA RR, composes the EvgS/EvgA TCS (Utsumi et al., 1994). This system is widely conserved in *E. coli* and *Shigella* (Johnson et al., 2014). A homologous system is found in *Bordetella pertussis* (the etiologic agent of whooping cough) as BvgS/BvgA, which controls a variety of virulence determinants (Arico et al., 1989; Uhl and Miller, 1994), and in *Klebsiella pneumoniae* as KvgS/KvgA, which is present only in the virulent strains (Lai et al., 2003; Lin et al., 2006). However, EvgS/EvgA is not found in any of the other sequenced bacteria. In *E. coli*, EvgS/EvgA upregulates a network of acid resistance genes, through a cascade of EvgA-YdeO-GadE regulators (Masuda and Church, 2003; Itou et al., 2009; De Biase and Lund, 2015), and via the SafA-PhoQ/PhoP-IraM-RpoS network, which involves two TCSs, two small TCS-connecting proteins, and a sigma factor (Eguchi et al., 2007, 2011). This upregulation of acid resistance genes confers severe acid resistance to cells in their exponential phase (Ma et al., 2004; Itou et al., 2009; Burton et al., 2010; De Biase and Lund, 2015). EvgS/EvgA also induces the expression of the EmrKY efflux pump via EvgA, which directly binds to the promoter region of the *emrKY* operon (Kato et al., 2000). The EmrKY pump belongs to the major facilitator superfamily and contributes to bile acid resistance (Nishino and Yamaguchi, 2001). Mutations affecting *emrK* or *emrY* gene cause a hypersensitive phenotype to the lethal effect of nalidixic acid, mitomycin C, ultraviolet irradiation, and hydrogen peroxide, suggesting that EmrKY might be pumping out toxic metabolites induced by DNA damage (Han et al., 2010). A recent report highlighted that this pump contributes to the survival of *Shigella flexneri* (the etiological agent of bacillary dysentery) within the host macrophage (Pasqua et al., 2019). This report also showed that *Shigella emrKY* is upregulated by EvgS/EvgA within host cells. There are other reports connecting EvgS/EvgA to *E. coli* pathogenicity. In enteropathogenic *E. coli*, expression of recombinant EvgA repressed the expression of the type III secretion system, which delivers a set of effector proteins into the host cell cytoplasm (Nadler et al., 2006). Another study showed that in avian pathogenic *E. coli*, an *evgS* mutant showed attenuated lung colonization in turkeys (Dziva et al., 2013).

EvgS is one of five unorthodox HK sensors in *E. coli* and has a large periplasmic domain with two tandem Venus flytrap domains (Sen et al., 2017). The cytoplasmic region consists of three catalytic domains: a HK domain, an intermediate receiver domain, and a Hpt (histidine-containing phosphotransfer) transmitter domain. Upon activation, the conserved histidine residue in the HK domain is autophosphorylated, and the phosphate is subsequently transferred to the aspartate residue of the intermediate receiver domain, then to the histidine residue of the Hpt transmitter (Perraud et al., 1998). This three-step phosphoryl-transfer reactions within the EvgS dimer is carried out in a *cis-cis-cis* mode, which is different from two other unorthodox HK sensors, ArcB (*cis-trans-trans*) and BarA (*trans-trans-trans*) (Kinoshita-Kikuta et al., 2016). From the Hpt transmitter, the phosphate is transferred to the aspartate residue of EvgA. The cytoplasmic linker region, between the transmembrane and HK domains, resembles a PAS domain, which is known to act as a molecular sensor responsive to signals such as oxygen, light, and voltage. Previous studies have shown that mildly acidic pH serves as an activating stimulus for EvgS (Ma et al., 2004; Burton et al., 2010; Eguchi and Utsumi, 2014). This signal is consistent with this system, which induces the expression of genes that confer severe acid resistance (De Biase and Lund, 2015). However, the mildly acidic medium needs to be a synthetic minimal medium, and not a rich medium such as LB (Lysogeny broth). Moreover, at least 150 mM of alkali metals must be included in the acidic medium for EvgS activation (Eguchi and Utsumi, 2014). The periplasmic region of EvgS is necessary for signal perception (Eguchi and Utsumi, 2014; Johnson et al., 2014), as well as the cytoplasmic PAS domain (Eguchi and Utsumi, 2014). Another report suggested that the PAS domain responds to acidic pH (Sen et al., 2017). In a different study, it has been shown that there is a natural variation in the pH-sensing capacity of EvgS, with some strains maintaining the ability to confer acid resistance despite being non-responsive to acidic pH (Roggiani et al., 2017). These reports suggest that there may be more input signals for EvgS.

The ArcB HK sensor of the ArcBA TCS is another member of the five unorthodox HK sensors of *E. coli* that responds to the changing respiratory conditions of growth. In *E. coli*, three dominant quinone types are found, which are active in facilitating electron transfer in the respiratory chain. These are ubiquinone (UQ), demethylmenaquinone (DMK), and menaquinone (MK). While DMK and MK are abundant during anaerobic conditions, UQ is the most abundant redox carrier during aerobic growth (Sharma et al., 2012). Under aerobic growth, these quinones silence the kinase activity of ArcB by oxidizing two redox-active cysteine residues in the cytoplasmic PAS domain that form interprotomer disulfide bonds in the ArcB dimer. Upon a shift from aerobic growth conditions to anaerobic, reduced forms of these quinones break the disulfide bonds and activate ArcB kinase activity (Georgellis et al., 2001; Malpica et al., 2004; Bekker et al., 2010; Sharma et al., 2012; Alvarez et al., 2013; van Beilen and Hellingwerf, 2016). Since both BvgS of *B. pertussis* and EvgS are unorthodox HK sensors and have cytoplasmic PAS domains as in ArcB, the effect of electron carriers on the kinase activities of the cytoplasmic soluble forms of EvgS and BvgS had been examined by Bock and Gross. In their study, oxidized UQ-0 (soluble analog of UQ-8), but not menadione (MK-3, soluble analog of MK-8), strongly inhibited the kinase activities of BvgS and EvgS (Bock and Gross, 2002). However, these *in vitro* studies were carried out using truncated soluble forms of HK. Whether the respiratory growth conditions affect EvgS activity in *E. coli* cells has not been examined yet. Therefore, in the present study, we examined *in vivo* EvgS activation under different respiratory conditions and found that EvgS activation only occurred under oxidative conditions. We propose oxidation as another stimulus perceived by the EvgS sensor.

## Materials and Methods

### Strains and Plasmids

The *E. coli* strains and plasmids used in this study are listed in Table 1.

**TABLE 1:** Strains and plasmids used in this study.

Strains or plasmids	Description	Reference or source
**Strains**		
MG1655	Wild type	Blattner et al., 1997
MG1655 *ydeP-lacZ*	MG1655 *ydeP-lacZY*	Eguchi and Utsumi, 2014
MG1655 *emrKY-lacZ*	MG1655 *emrKY-lacZY*	Eguchi and Utsumi, 2014
MG1655 *lacZ*	MG1655 *lacZ:cat*	This study
MG1655 *lacZ ydeP-lacZ*	MG1655 *lacZ:cat ydeP-lacZY*	This study
MG1655 *lacZ emrKY-lacZ*	MG1655 *lacZ:cat emrKY-lacZY*	This study
JW3901	BW25113 *menA:kan*	Keio collection (Baba et al., 2006)
MG1601	MC4100 *mgtA:*λp*lac*Mu55	Kato et al., 1999
MG1655 *lacZ menA ydeP-lacZ*	MG1655 *lacZ:cat menA ydeP-lacZY*	This study
MU1227	W3110 *polA^*TS*^ rha lac Str^*r*^ ubiA:cat*	Suzuki et al., 1994
MG1655 *ubiA ydeP-lacZ*	MG1655 *ubiA:cat ydeP-lacZY*	This study
MG1655 *evgS ydeP-lacZ*	MG1655 *evgS:cat ydeP-lacZY*	Eguchi and Utsumi, 2014

**Plasmids**		
pBAD18	Cloning vector with an arabinose promoter	Guzman et al., 1995
pBADevgA	*evgA* cloned downstream of an arabinose promoter	This study
pBADevgS	*evgS* cloned downstream of an arabinose promoter	Eguchi and Utsumi, 2014
pBADevgS C663A	pBADevgS with an EvgS C663A mutation	This study
pBADevgS C671A	pBADevgS with an EvgS C671A mutation	This study
pBADevgS C683A	pBADevgS with an EvgS C683A mutation	This study
pBADevgS C671A C683A	pBADevgS with EvgS C671A C683A mutations	This study
pBADevgS-cyt	*evgS553-1197* cloned downstream of an arabinose promoter, expresses EvgS553-1197	This study
pBADevgS C671S	pBADevgS with an EvgS C671S mutation	This study
pBADevgS C683S	pBADevgS with an EvgS C683S mutation	This study
pBADevgS C671M	pBADevgS with an EvgS C671M mutation	This study
pBADevgS C683M	pBADevgS with an EvgS C683M mutation	This study
pBADubiA	*ubiA* cloned downstream of an arabinose promoter	This study

### Growth Conditions

Cultures were grown in LB medium (1% w/v tryptone, 0.5% w/v yeast extract, 1% w/v NaCl, pH 7.5) at 37°C with shaking at 180 rpm, unless stated otherwise. Activation of the EvgS/EvgA system was carried out using M9 medium supplemented with 100 mM 2-(*N*-morpholino)ethanesulfonic acid (MES) and 100 mM KCl, with the pH adjusted to 5.7 using HCl.

When necessary, selective antibiotics (100 μg/mL ampicillin, 25μg/mL kanamycin, or 25 μg/mL chloramphenicol) were added to the medium. Arabinose was added to the culture at a final concentration of 1% w/v for EvgA overproduction and 0.02% for expression of EvgS and its variants. For *ubiA* mutants, 1 mM uracil was added to the medium to accelerate growth (personal communication).

### Construction of Reporter Strains

The primers used in this study are listed in Table 2. To decrease the background activity of the *lacZ*-based reporter strains, *lacZ* of strain MG1655 was disrupted by inserting a chloramphenicol resistance cassette, according to a one-step inactivation method (Datsenko and Wanner, 2000), using primers lacZ-P2-F and lacZ-P1-R. The *lacZ* deletion was transferred to reporter strains MG1655 *ydeP-lacZ* and MG1655 *emrKY-lacZ* by P1 transduction to obtain MG1655 *lacZ ydeP-lacZ* and MG1655 *lacZ emrKY-lacZ*, respectively. Deletion of *menA* was transferred from JW3901 to MG1655 by P1 transduction, followed by removal of the kanamycin resistance cassette with pCP20 plasmid (Datsenko and Wanner, 2000), insertion of *ydeP-lacZ*, and deletion of *lacZ* by P1 transduction from MG1655 *ydeP-lacZ* and MG1655 *lacZ*, respectively. Deletion of *ubiA* was transferred from MU1227 to MG1655 *ydeP-lacZ* by P1 transduction.

**TABLE 2:** Primers used in this study.

Primers	Sequence (5′–3′)
lacZ-P2-F	TTATGCTTCCGGCTCGTATGTTGTGTGGAATTGTGA GCGGCATATGAATATCCTCCTTAG
lacZ-P1-R	ATGGATTTCCTTACGCGAAATACGGGCAGACATGGC CTGCGTGTAGGCTGGAGCTGCTTC
evgA-*Nco*I-F	ATCATGCCATGGGCAACGCAATAATTATTGATG
evgA-*Xho*I-R	ATCCGCTCGAGGCCGATTTTGTTACGTTGTG
EvgS-cyt-F	TGGGGATTCTACCTGTTACG
EvgS-cyt-R	CATGGGTATGTATATCTCCTTC
EvgS C663A-F	CATCGAGAAAAGAGCCATTAATCACTGGCATAC
EvgS C663A-R	GTATGCCAGTGATTAATGGCTCTTTTCTCGATG
EvgS C671A-F	CTGGCATACATTAGCCAATCTTCCTGCAAG
EvgS C671A-R	CTTGCAGGAAGATTGGCTAATGTATGCCAG
EvgS C683A-F	CAATGCAGTATATATTGCTGGTTGGCAAGATATTAC
EvgS C683A-R	GTAATATCTTGCCAACCAGCAATATATACTGCATTG
EvgS C671S-F	CTGGCATACATTATCCAATCTTCCTGCAAG
EvgS C671S-R	CTTGCAGGAAGATTGGATAATGTATGCCAG
EvgS C683S-F	CAATGCAGTATATATTTCTGGTTGGCAAGATATTAC
EvgS C683S-R	GTAATATCTTGCCAACCAGAAATATATACTGCATTG
EvgS C671M-F	CTGGCATACATTAATGAATCTTCCTGCAAG
EvgS C671M-R	CTTGCAGGAAGATTCATTAATGTATGCCAG
EvgS C683M-F	CAATGCAGTATATATTATGGGTTGGCAAGATATTAC
EvgS C683M-R	GTAATATCTTGCCAACCCATAATATATACTGCATTG
ubiA-*Nco*I-F	ATCATGCCATGGAGTGGAGTCTGACGCAG
ubiA-*Xho*I-R	ATCCGCTCGAGGAAATGCCAGTAACTCATTGC

**Sequencing primers**	
pBAD-F	ATGCCATAGCATTTTTATCC
pBAD-R	TGATTTAATCTGTATCAGGC
EvgS-R1	TGCACACCATCAGTGGCTTC
EvgS-R2	ACTGCTGCAACTTAATGC
EvgS-R3	TGTGACTTCATGCGCATTAG
EvgS-R4	ACCAGAGCATCAAGTTCAC
EvgS-R5	TCATGTTCAGTGAGTTCTAATGG

*Restriction sites are shown in red.*

### Construction of Expression Plasmids

Plasmids for expressing *evgA* and *ubiA* were constructed by PCR amplified DNA fragments with the primer pairs, evgA-*Nco*I-F + evgA-*Xho*I-R and ubiA-*Nco*I-F + ubiA-*Xho*I-R, and ligating them to *Nco*I- and *Xho*I- (Toyobo, Osaka, Japan) digested pBAD vector. To construct plasmids for expressing EvgS variants, site-directed mutagenesis of C663A, C671A, C683A, C671S, C683S, C671M, and C683M was performed using the primers listed in Table 2 (sites of mutation are underlined), PrimeSTAR^®^ HS DNA polymerase (Takara Bio, Kusatsu, Japan), and pBADevgS plasmid. The PCR product was treated with *Dpn*I (Toyobo) to degrade the template plasmid and transformed into DH5α. Site-directed mutagenesis was also performed to create plasmids for the double mutant C671A C683A. The plasmid for expressing the EvgS cytoplasmic region (553–1197), pBADevgS-cyt, was constructed using EvgS-cyt-F and EvgS-cyt-R primers, PrimeSTAR^®^ HS DNA polymerase, and pBADevgS as the template for PCR. The PCR product was treated with *Dpn*I, phosphorylated at its 5′ end with polynucleotide kinase (Toyobo), and self-ligated. All plasmids were confirmed using DNA sequencing.

### Reporter Assay

A single colony of an *E. coli* strain was inoculated in 10 mL of LB medium containing appropriate antibiotics and grown overnight with shaking at 37°C. This culture was diluted 100-fold with 10 mL of LB (ampicillin added for transformants) and grown at 37°C with shaking to an optical density at 660 nm (OD_660_) of 0.6. Three 10 mL cultures of each strain for each sampling time were prepared for the assay. Cells were collected using centrifugation (2,300 × *g*, 10 min, room temperature) and resuspended in 10 mL of M9 medium (pH 5.7) supplemented with 100 mM MES and 100 mM KCl (EvgS-activation medium). This cell suspension in EvgS-activation medium was allocated in the following three ways. Aerobic condition: 500 μL of the cell suspension was placed into glass test tubes (φ13 mm × 100 mm) with aluminum caps and further shaken (180 rpm) at 37°C. Semi-aerobic condition: 500 μL of the cell suspension was placed into the same glass test tubes with aluminum caps as for aerobic condition, and stood at 37°C. Anaerobic condition: screw-cap tubes were filled up to the rim with the cell suspension (approximately 9.5 mL) and stood at 37°C. At appropriate sampling times, the cultures were subjected to β-galactosidase assays (performed in duplicate), the results for which were expressed in Miller units (Miller, 1972). The data shown are mean and standard deviation of the results from at least three biologically separate cultures. Statistical analyses were performed by Dunnett’s multiple comparisons test with Prism software (version 7.02) (GraphPad, La Jolla, United States), using the time 0 sample as the control.

### Detection of EvgA, EvgS, and EvgS Variants

One milliliter of the cell culture used for the reporter assays was centrifuged (13,000 × *g*, 3 min, 4°C) and the obtained pellet was resuspended in 1 mL of saline. The OD_600_ of each suspension was measured and adjusted with saline to obtain equal cell density. Protein denaturation of 500 μL of the adjusted cell suspension was performed by adding 500 μL of 10% trichloroacetic acid (TCA), vortexed, and placed on ice for 20 min. The denatured proteins were precipitated using centrifugation (17,800 × *g*, 15 min, 4°C). The obtained pellets were washed with 500 μL of acetone followed by centrifugation (17,800 × *g*, 15 min, 4°C) in order to remove the residual TCA. The pellets were dissolved in 100 μL of 1 × sample buffer for SDS-PAGE, and heated for 5 min at 95°C or for 30 min at 37°C for membrane proteins. Twenty microliters of each sample was subjected to SDS-PAGE and the electrophoresed proteins were transferred to a polyvinylidene difluoride membrane (Immun-Blot^®^ PVDF, Bio-Rad, Hercules, United States). EvgA (His-tagged at the C-terminal end) was probed with an anti-6X His tag antibody (Abcam, Cambridge, United Kingdom), while EvgS and its variants were probed with anti-EvgS antiserum (Eguchi and Utsumi, 2014). Detection was carried out using goat anti-rabbit horseradish peroxidase-linked IgG (Abcam) and Immobilon^TM^ Western Chemiluminescent HRP Substrate (Merck Millipore, Burlington, United States). Signals were acquired using a MultiImager II Multibox (BioTools, Maebashi, Japan).

## Results

### Aeration Is Required for EvgS Activation

**FIGURE 1:**
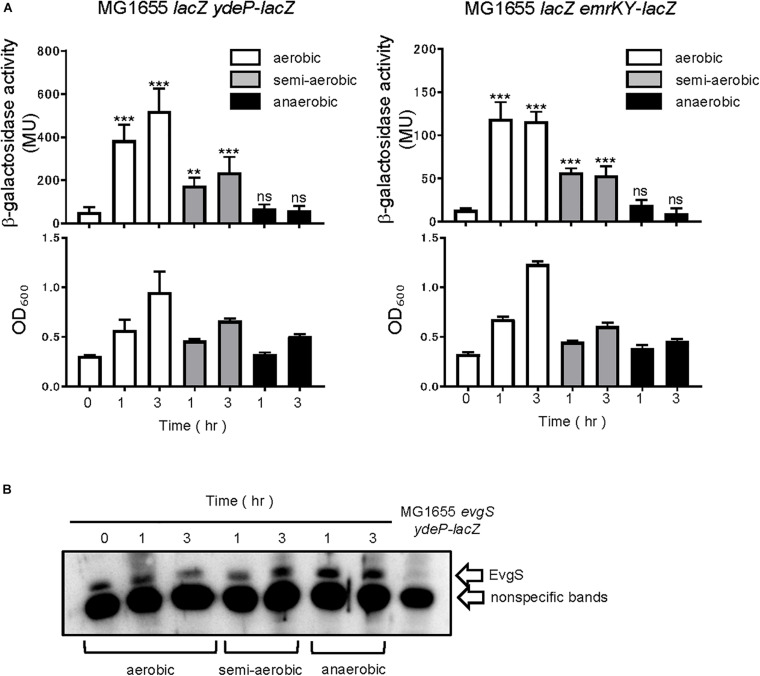
Aeration is required for EvgS/EvgA activation. **(A)** Activity of the *ydeP-lacZ* and *emrKY-lacZ* reporters in different culturing conditions (upper panels). Cells were grown in EvgS-activation medium under aerobic (white bars), semi-aerobic (gray bars), or anaerobic condition (black bars) at 37°C. Cell growth indicated by optical density at 600 nm is shown in the lower panels. Data represent the average of three biologically independent replicates. Error bars indicate the standard deviation, and statistical analyses of each redox condition group were performed using Dunnett’s multiple comparison test with the time 0 sample as the control. ns, not significant; **0.001 ≤ *p* < 0.01; ***p < 0.001. **(B)** EvgS expression in MG1655 *lacZ ydeP-lacZ*. Immunoblotting analysis using anti-EvgS antiserum for EvgS detection is shown. Samples are from the same culture as those subjected to reporter assays. Lane at the right end shows the sample from an *evgS* deleted strain.

The EvgS/EvgA system is activated under mildly acidic conditions, in the presence of alkali metals (Eguchi and Utsumi, 2014). Since the EvgS sensor has a cytoplasmic PAS domain adjacent to the membrane, we examined whether respiratory growth conditions affect EvgS activity *in vivo*. Three conditions were compared in this study: aerobic (shaking at 180 rpm), semi-aerobic (standing), and anaerobic (standing screw-capped tube filled with cell culture). The EvgS/EvgA reporter strain, MG1655*lacZ ydeP-lacZ*, with a *lacZ* insertion immediately downstream of the *ydeP* gene and deletion of chromosomal *lacZ*, was grown to the exponential phase in an EvgS-inactivating medium (LB), followed by exchange to an EvgS-activation medium (M9 + MES, pH 5.7), and further incubated at 37°C under aerobic, semi-aerobic, and anaerobic conditions. For each sample, β-galactosidase activity was measured to examine how EvgS/EvgA responded to the different respiratory conditions. As shown in Figure 1A, EvgS/EvgA was activated after 1 and 3 h of aerobic and semi-aerobic growth, with a lower level of activation in the latter condition. No EvgS/EvgA activation was observed under the anaerobic condition. To confirm that the *ydeP-lacZ* reporter activity represented the EvgS/EvgA activity, and was not influenced by other factors that may respond to the anaerobic condition and shut down *ydeP-lacZ* expression, we performed the same experiment using the MG1655*lacZ emrKY-lacZ* reporter strain, which contained a *lacZ* insertion immediately downstream of the *emrKY* operon and deletion of chromosomal *lacZ*. The *emrKY* operon and *ydeP* are both directly regulated by the RR EvgA (Kato et al., 2000; Itou et al., 2009), and according to the EcoCyc database (Keseler et al., 2017), these two genes are not regulated by any other common transcriptional factors besides EvgA. As shown in Figure 1A, MG1655*lacZ emrKY-lacZ* also showed decreased activation under the semi-aerobic condition, and no activation under the anaerobic condition, clearly indicating that the EvgS/EvgA system responded to the availability of oxygen. We also examined EvgS expression in cells used in the reporter assays and found that EvgS levels were fairly constant among the different incubation conditions (Figure 1B, full gel results shown in Supplementary Figure 1). This is in line with our previous results, which suggested that the expression of the *evgAS* operon is not autoregulated by the EvgS/EvgA system (Eguchi et al., 2003).

**FIGURE 2:**
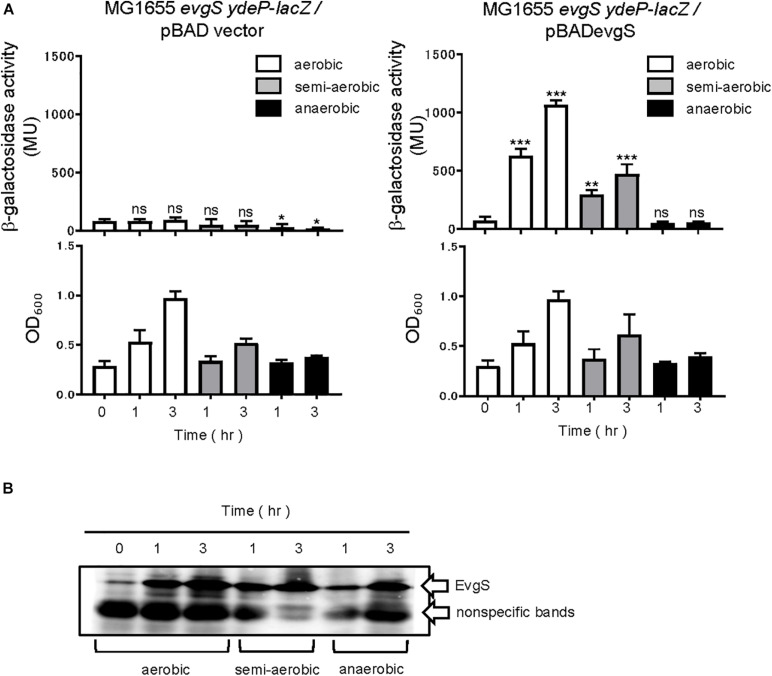
Response to aeration is EvgS-dependent. **(A)** Activity of the *ydeP-lacZ* reporter in different culturing conditions (upper panels). Cells were grown in EvgS-activation medium with 0.02% arabinose under aerobic (white bars), semi-aerobic (gray bars), or anaerobic condition (black bars) at 37°C. Cell growth indicated by optical density at 600 nm is shown in the lower panels. Data represent the average of three biologically independent replicates. Error bars indicate the standard deviation, and statistical analyses of each redox condition group were performed as described in Figure 1. ns, not significant; *0.01 ≤ *p* < 0.05; **0.001 ≤ *p* < 0.01; ***p < 0.001. **(B)** EvgS expression in MG1655 *evgS ydeP-lacZ/*pBADevgS. Immunoblotting analysis using anti-EvgS antiserum for EvgS detection is shown. Samples are from the same culture as those subjected to reporter assays.

Next, we checked whether the activity estimated by our reporter assays was dependent on EvgS. Deleting *evgS* resulted in no activation in the EvgS-activation medium, while expressing EvgS using an EvgS-expressing plasmid (pBADevgS) rescued the activation (Figure 2A). Activation was also lost under the anaerobic condition. The small decrease in β-galactosidase activity under anaerobic condition in MG1655 *evgS ydeP-lacZ*/pBAD vector may be due to the repression of the chromosomal *lacZ* expression. We confirmed EvgS expression under all conditions (Figure 2B, full gel results shown in Supplementary Figure 2).

Since the anaerobic condition retarded cell growth, it is possible that the decreased cell activity affected *lacZ* expression, and thus, lowered the β-galactosidase activity. To confirm this, we tested another *E. coli* reporter strain with a different two-component system, PhoQ/PhoP. This reporter strain, MG1601 (Kato et al., 1999), measures the promoter activity of *mgtA*, a component of the PhoP regulon. When this strain was first grown until the exponential phase in PhoQ-inactivating medium (LB + 20 mM MgSO_4_), followed by exchange to a PhoQ-activating medium (LB), the PhoQ/PhoP system showed activation, regardless of the difference in the respiratory conditions (aerobic, semi-aerobic, or anaerobic) (Supplementary Figure 3). Thus, *lacZ* was still expressed during reduced growth under the anaerobic condition, and EvgS/EvgA inactivation in this condition was not due to growth retardation.

**FIGURE 3:**
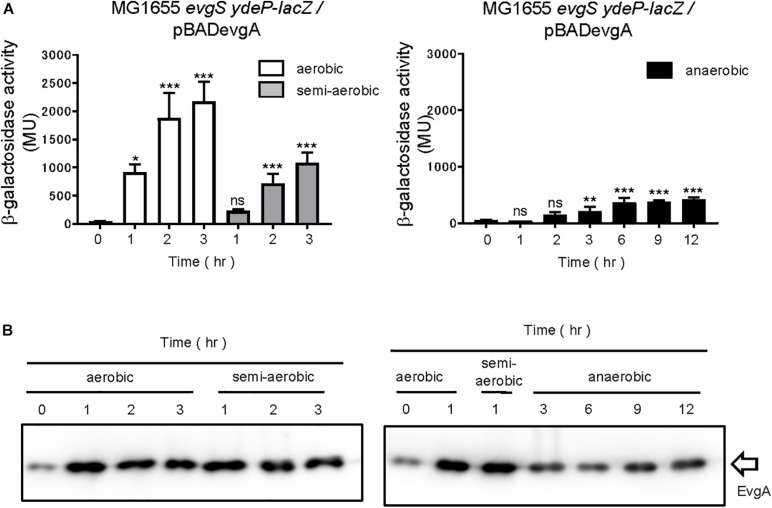
Aeration is not required for activating EvgS/EvgA by EvgA-overproduction. **(A)** Activity of the *ydeP-lacZ* reporter by inducing EvgA from pBADevgA. Cells were grown in EvgS-activation medium with 1% arabinose under aerobic (white bars), semi-aerobic (gray bars), or anaerobic condition (black bars) at 37°C. Optical density at 600 nm of the cell cultures subjected to reporter assays is shown in Supplementary Figure 4. Data represent the average of three biologically independent replicates. Error bars indicate the standard deviation, and statistical analyses of each redox condition group were performed as described in Figure 1. ns, not significant; *0.01 ≤ *p* < 0.05; **0.001 ≤ *p* < 0.01; ***p < 0.001. **(B)** EvgA expression in MG1655 *evgS ydeP-lacZ/*pBADevgA. Immunoblotting analysis using anti-His6 antibody for EvgA-His detection is shown. Samples are from the same culture as those subjected to reporter assays.

Furthermore, we examined whether the change in respiratory conditions was sensed by the sensor EvgS or by the RR EvgA. Overexpression of RR is often accompanied by the activation of its TCS, which is also true with the overexpression of EvgA (Nishino and Yamaguchi, 2001). RRs may be phosphorylated by small-molecule phosphate donors such as acetyl phosphate. In some cases, increased expression of regulon components due to RR overexpression can take place even in the absence of RR phosphorylation (Bekker et al., 2010). We overexpressed EvgA (His-tagged at the C-terminal) from a pBADevgA plasmid in an *evgS*-deleted reporter strain, MG1655*evgS ydeP-lacZ*. Arabinose was added to the EvgS-activation medium at time 0 for EvgA induction, which was confirmed using immunoblotting for the His tag of EvgA. EvgS/EvgA activation was observed under aerobic and semi-aerobic conditions as well as under the anaerobic condition (Figure 3A). Activation of EvgS/EvgA under the anaerobic condition was slow and weak compared to its activation under the aerobic and semi-aerobic conditions. EvgA induction occurred slowly under the anaerobic condition, possibly due to retarded translational activity (Figure 3B, full gel results shown in Supplementary Figure 5) and corresponded to EvgS/EvgA activity. This shows that EvgS/EvgA activation by EvgA overproduction can be performed under anaerobic conditions, which is not what we observed in the reporter assays in Figure 1. Consequently, we claim that only the sensor EvgS, but not EvgA, responds to the change in the redox state, and that oxidation is required for EvgS activation.

### The Two Cysteines in the PAS Domain Are Involved in Response to the Anaerobic State

**FIGURE 4:**
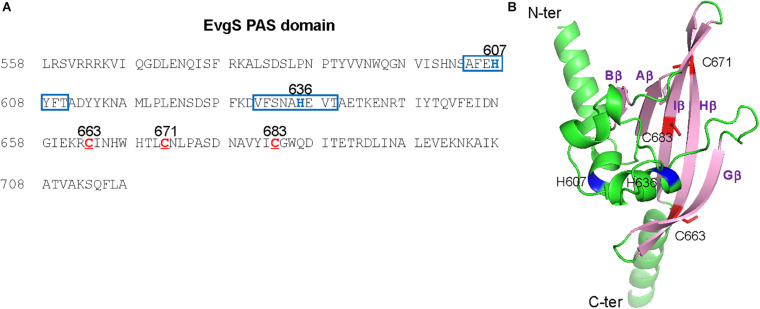
Positions of three cysteine residues and putative quinone-binding motifs in the EvgS PAS domain. **(A)** The sequence of the EvgS PAS domain is shown with three cysteines (C663, C671, and C683) depicted in red. In addition, the putative quinone-binding motifs are boxed in blue with central histidine residues (H607 and H636) depicted in blue. **(B)** A homology model of the EvgS PAS domain was created by the SWISS-MODEL homology-modeling server (https://swissmodel.expasy.org, Waterhouse et al., 2018) using the PAS domain of BvgS of *Bordetella pertussis* (PDB entry ID: 6ZJ8) as a template. The model is presented by a cartoon model with C663, C671 in Hβ strand, and C683 in Iβ strand depicted in red. Side chains of C663, C671, and C683 are shown with sticks. The central histidine residues, H607 and H636, located on two Q-helices, are depicted in blue.

**FIGURE 5:**
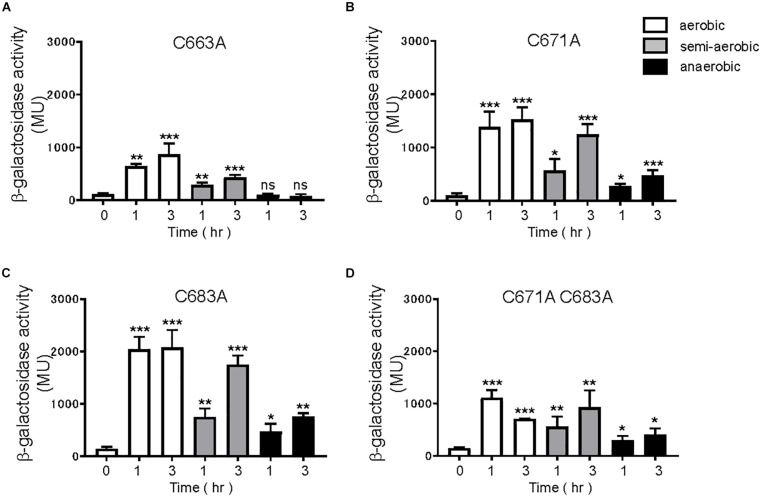
Response to aeration in EvgS-PAS mutants. **(A)** MG1655 *evgS ydeP-lacZ/*pBADevgS C663A, **(B)** MG1655 *evgS ydeP-lacZ/*pBADevgS C671A, **(C)** MG1655 *evgS ydeP-lacZ/*pBADevgS C683A, **(D)** MG1655 *evgS ydeP-lacZ/*pBADevgS C671A C683A. Activity of the *ydeP-lacZ* reporter in different culturing conditions (upper panels). Cells were grown in EvgS-activation medium with 0.02% arabinose under aerobic (white bars), semi-aerobic (gray bars), or anaerobic condition (black bars) at 37°C. Optical density at 600 nm of the cell cultures subjected to reporter assays is shown in Supplementary Figure 6. Data represent the average of three biologically independent replicates. Error bars indicate the standard deviation, and statistical analyses of each redox condition group were performed as described in Figure 1. ns, not significant; *0.01 ≤ *p* < 0.05; **0.001 ≤ *p* < 0.01; ***p < 0.001.

EvgS is an unorthodox HK sensor, similar to the anaerobic sensor ArcB. ArcB senses the redox state at two cysteines positioned at 180 and 241 within its cytoplasmic PAS domain (Malpica et al., 2004). We searched for cysteine residues in the PAS domain of EvgS and found three cysteines at the positions 663, 671, and 683 (Figure 4A). We created a homology model of the EvgS PAS domain monomer by the SWISS-MODEL homology-modeling server^1^ (Waterhouse et al., 2018) using the PAS domain of BvgS of *Bordetella pertussis* (PDB entry ID: 6ZJ8) as a template (Figure 4B). According to this model, C663 and C671 are in Hβ strand, and C683 in Iβ strand. Alanine mutations were made for each of these cysteines in pBADevgS to express EvgS C663A, EvgS C671A, and EvgS C683A. These EvgS mutants were expressed in an *evgS*-deleted reporter strain and assayed under conditions similar to those of wild-type EvgS (Figure 2). When compared to the wild-type, EvgS C663A showed similar EvgS/EvgA activity under all the respiratory conditions, with the anaerobic condition shutting down EvgS/EvgA activity (Figure 5A). However, EvgS C671A and EvgS C683A showed enhanced EvgS/EvgA activation, compared to the wild-type, under the aerobic and semi-aerobic conditions. Although mutations within the PAS domain frequently cause a locked-on state of EvgS (Kato et al., 2000; Johnson et al., 2014), C671A and C683A were inactivated during growth in LB (Figures 5B,C, time 0) and were only activated upon transfer to the EvgS-activation medium. Both the mutants showed EvgS/EvgA activation even under the anaerobic condition, indicating that the shutdown of EvgS under anaerobic conditions is alleviated by the C671A and C683A mutations. The double mutant of EvgS (C671A C683A) also showed enhanced activation compared to the wild-type, but not as high as the individual C671A and C683A mutants (Figure 5D). Protein expression of EvgS mutants was confirmed in all reporter strains (Supplementary Figure 7). These results strengthen our hypothesis that EvgS senses the redox state.

**FIGURE 6:**
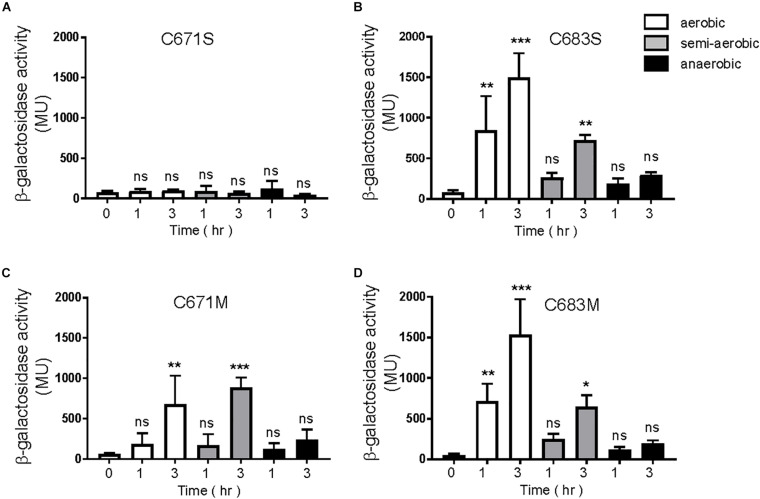
Response to aeration in EvgS-PAS mutants. **(A)** MG1655 *evgS ydeP-lacZ/*pBADevgS C671S; **(B)** MG1655 *evgS ydeP-lacZ/*pBADevgS C683S; **(C)** MG1655 *evgS ydeP-lacZ/*pBADevgS C671M; **(D)** MG1655 *evgS ydeP-lacZ/*pBADevgS C683M. Activity of the *ydeP-lacZ* reporter in different culturing conditions (upper panels). Cells were grown in EvgS-activation medium with 0.02% arabinose under aerobic (white bars), semi-aerobic (gray bars), or anaerobic condition (black bars) at 37°C. Optical density at 600 nm of the cell cultures subjected to reporter assays is shown in Supplementary Figure 8. Data represent the average of three biologically independent replicates. Error bars indicate the standard deviation, and statistical analyses of each redox condition group were performed as described in Figure 1. ns, not significant; *0.01 ≤ *p* < 0.05; **0.001 ≤ *p* < 0.01; ***p < 0.001.

A canonical PAS fold comprises of a five-stranded antiparallel β-sheet and several α-helices flanking the sheet (Möglich et al., 2009). According to our homology model of the EvgS PAS domain in Figure 4B, C671 and C683 are positioned in the β-strands Hβ and Iβ, respectively. The side chain of C671 protrudes outside the molecule, while the side chain of C683 faces inside the molecule. Thus, it is assumed that C671 and C683 cannot form intraprotomer disulfide bonds. When EvgS C671S was expressed in MG1655 *evgS ydeP-lacZ* strain, EvgS activity was lost under all conditions (Figure 6A). This may mimic the breakage of a disulfide bond between C671. However, when EvgS C671M was expressed, EvgS activity was observed under aerobic and semi-aerobic conditions and not under anerobic condition (Figure 6C). The difference in protein expression of EvgS C671S and C671M may also explain the reduced activity in EvgS C671S (Supplementary Figure 9). We also expressed EvgS C683S and C683M in MG1655 *evgS ydeP-lacZ* strain. Both EvgS variants showed EvgS activity under aerobic and semi-aerobic conditions, and not under anaerobic condition (Figures 6B,D). At present, we cannot propose a mechanism of how C671 and C683 are involved in the redox control of EvgS activity only from our results. Further investigation such as *in vivo* cross-linking and structural studies of the EvgS PAS domain is necessary for clarification. C671 and C683 are conserved among EvgS PAS domains of different *E. coli* strains and *Shigella* species (Supplementary Figure 10). Cysteines were not found at this position in the BvgS-PAS domain.

### Membrane Localization Is Necessary for Response to the Anaerobic Condition

**FIGURE 7:**
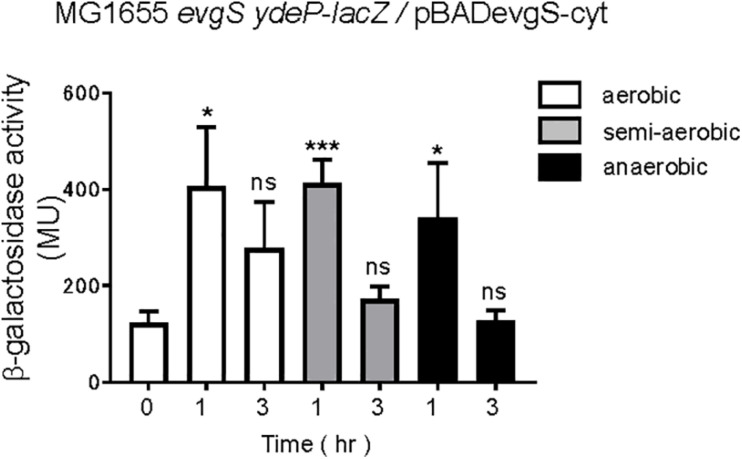
Aeration is not necessary for EvgS/EvgA activation in strains expressing the cytoplasmic region of EvgS (EvgS-Cyt). Activity of the *ydeP-lacZ* reporter in different culturing conditions (upper panel). Cells were grown in EvgS-activation medium with 0.02% arabinose under aerobic (white bars), semi-aerobic (gray bars), or anaerobic condition (black bars) at 37°C. Optical density at 600 nm of the cell cultures subjected to reporter assays is shown in Supplementary Figure 11. Data represent the average of three biologically independent replicates. Error bars indicate the standard deviation, and statistical analyses of each redox condition group were performed as described in Figure 1. ns, not significant; *0.01 ≤ *p* < 0.05; ***p < 0.001.

Expressing the cytoplasmic region of EvgS also activates the EvgS/EvgA system without EvgS-activating signals (Sen et al., 2017). We examined whether the cytoplasmic region of EvgS retained its response to the redox state. The cytoplasmic region of EvgS was expressed from the pBADevgS-cyt plasmid in an *evgS*-deleted reporter strain and assayed under conditions similar to those of wild-type EvgS (Figure 2). Expression of the cytoplasmic region of EvgS under aerobic conditions resulted in activation of the EvgS/EvgA system (Figure 7), as previously reported (Sen et al., 2017). This activation was also observed in the semi-aerobic and anaerobic cultures. We confirmed expression of the cytoplasmic region of EvgS under all conditions (Supplementary Figure 12). Although the cytoplasmic region of EvgS comprises the PAS domain, the results shown in Figure 7 indicate that the cytoplasmic region of EvgS did not respond to the redox state. Therefore, it is necessary for EvgS to be anchored to the membrane for responding to anaerobic conditions, suggesting that membrane association promotes the interaction between the cytoplasmic region of the sensor protein and the redox signal. In addition, the loss of the transmembrane domain, which maintains the proper distance between protomers of the linking cytoplasmic region, may prohibit the relevant conformational change required for EvgS inactivation.

### Ubiquinone Is Required for EvgS Activation

The anaerobic sensor, ArcB, utilizes oxidized and reduced forms of UQ, DMK, and MK to control ArcB activity (Georgellis et al., 2001; Bekker et al., 2010; Alvarez et al., 2013; Sharma et al., 2013; van Beilen and Hellingwerf, 2016). Since membrane localization of EvgS was required for sensing the redox condition, we examined whether these three dominant quinones in *E. coli* control EvgS activity.

**FIGURE 8:**
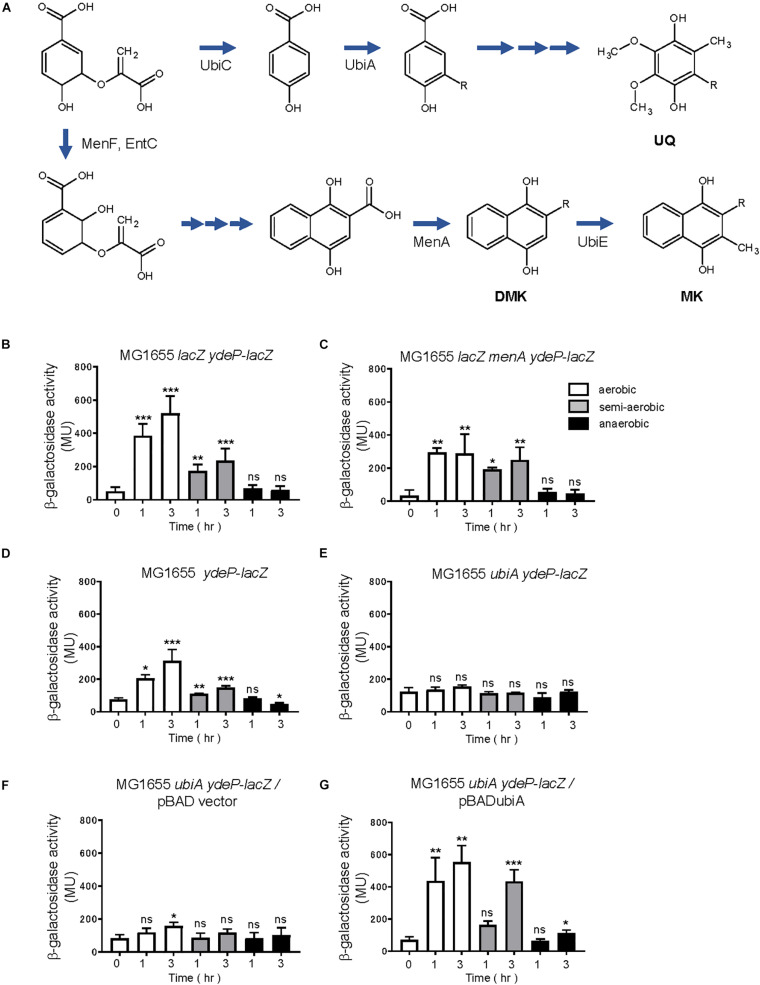
Ubiquinone is required for EvgS/EvgA activation. **(A)** Biosynthesis of ubiquinone (UQ), demethylmenaquinone (DMK), and menaquinone (MK) in *E. coli* (arranged from reference, van Beilen and Hellingwerf, 2016). **(B–G)** Activity of the *ydeP-lacZ* reporter in different culturing conditions (upper panels). Cells were grown in EvgS-activation medium with 0.02% arabinose under aerobic (white bars), semi-aerobic (gray bars), or anaerobic condition (black bars) at 37°C. Uracil was added to the medium at a concentration of 1 mM for experiments **(D–G)**. Optical density at 600 nm of the cell cultures subjected to reporter assays is shown in Supplementary Figure 13. Data represent the average of three biologically independent replicates. Error bars indicate the standard deviation, and statistical analyses of each redox condition group were performed as described in Figure 1. ns, not significant; *0.01 ≤ *p* < 0.05; **0.001 ≤ *p* < 0.01; ***p < 0.001. **(B)** MG1655 *lacZ ydeP-lacZ*, **(C)** MG1655 *lacZ menA ydeP-lacZ* (no DMK/MK), **(D)** MG1655 *ydeP-lacZ*, **(E)** MG1655 *ubiA ydeP-lacZ* (no UQ), **(F)** MG1655 *ubiA ydeP-lacZ/*pBAD vector (no UQ), **(G)** MG1655 *ubiA ydeP-lacZ/*pBADubiA (with UQ).

To construct a *ydeP-lacZ* reporter strain without DMK and MK, *menA*, which encodes 1,4-dihydroxy-2-naphthoate octaprenyltransferase, was deleted from the reporter strain. Deletion of this gene blocks DMK and MK biosynthesis (Figure 8A). When MG1655 *lacZ menA ydeP-lacZ* was assayed, EvgS was found to be activated under the aerobic and semi-aerobic conditions, but not under the anaerobic condition, as seen in case of wild-type MG1655 *lacZ ydeP-lacZ* (Figures 8B,C). This indicates that both DMK and MK are not essential for EvgS to respond to the redox state. However, the small decrease in EvgS activity under aerobic condition in MG1655 *lacZ menA ydeP-lacZ* suggests involvement of these two quinones for EvgS activation under aerobic condition.

For construction of a reporter strain without UQ, *ubiA*, which encodes 4-hydroxybenzoate octaprenyltransferase, was deleted from the reporter strain. Deletion of this gene hinders UQ biosynthesis (Figure 8A), resulting in retarded cell growth. Uracil, at a concentration of 1 mM, was added to the medium to support cell growth for the assay (personal communication). In contrast to the *menA*-deleted strain, deletion of *ubiA* resulted in no activation of EvgS (Figures 8D,E) under any of the redox conditions. Expression of *ubiA* from a UbiA-expressing plasmid, pBADubiA, complemented the *ubiA* deletion (Figures 8F,G), suggesting that UQ is required for the oxidative activation of EvgS.

Finally, we expressed EvgS, EvgS C671A, EvgS C683A, and EvgS C671A C683A in the *ubiA*-deleted reporter strain, MG1655 *ubiA ydeP-lacZ* (the host strain retains its *evgS* gene). To our surprise, additional expression of the wild type EvgS showed EvgS activity under aerobic and semi-aerobic state, but not under anaerobic state, as seen in the *evgS* complemented strain in Figure 2A. This indicates that when EvgS is overexpressed, EvgS activation can be controlled without UQ, presumably by DMK and MK. Oxidized UQ may have higher affinity against EvgS than oxidized DMK and MK. When EvgS C671A, EvgS C683A, and EvgS C671A C683A were each expressed in MG1655 *ubiA ydeP-lacZ*, enhanced level of EvgS activation was also observed under all redox condition. When this result was compared with that in the presence of UQ (Figure 5), higher EvgS activity was found under anaerobic conditions. This suggests that reduced form of UQ may be required for the repression of the activity of EvgS variants. Protein expression of EvgS and EvgS variants was confirmed in all reporter strains. Expression of EvgS and EvgS C671A was weaker than EvgS C683A and EvgS C671A C683A from unknown reasons (Supplementary Figure 15).

## Discussion

**FIGURE 9:**
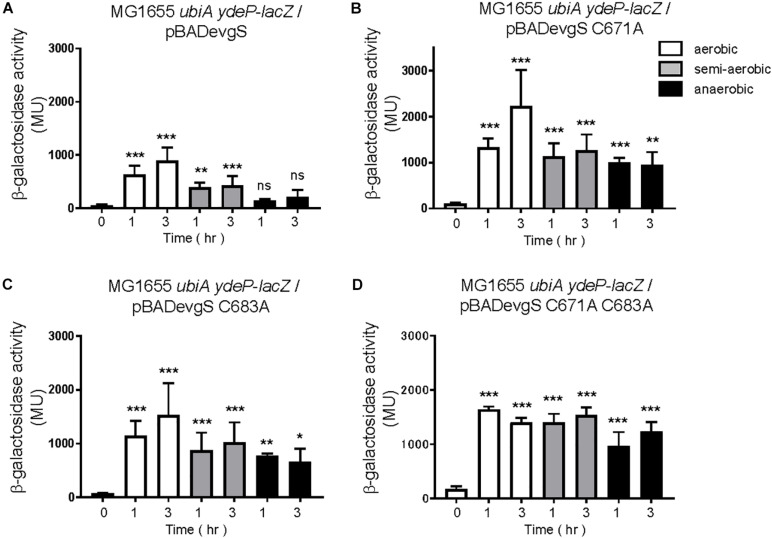
Ubiquinone is not essential for EvgS/EvgA activation in strains overexpressing EvgS and EvgS-PAS mutants. **(A–D)** Activity of the *ydeP-lacZ* reporter in different culturing conditions (upper panel). Cells were grown in EvgS activation-medium with 0.02% arabinose and 1 mM uracil under aerobic (white bars), semi-aerobic (gray bars), or anaerobic condition (black bars) at 37°C. Optical density at 600 nm of the cell cultures subjected to reporter assays is shown in Supplementary Figure 14. Data represent the average of three biologically independent replicates. Error bars indicate the standard deviation, and statistical analyses of each redox condition group were performed as described in Figure 1. ns, not significant; *0.01 ≤ *p* < 0.05; **0.001 ≤ *p* < 0.01; ***p < 0.001. **(A)** MG1655 *ubiA ydeP-lacZ/*pBADevgS, **(B)** MG1655 *ubiA ydeP-lacZ/*pBADevgS C671A, **(C)** MG1655 *ubiA ydeP-lacZ/*pBADevgS C683A, **(D)** MG1655 *ubiA ydeP-lacZ/*pBADevgS C671A C683A.

In the present *in vivo* study using an EvgS/EvgA reporter strain, we have shown that activation of EvgS requires a ubiquinone-dependent oxidative condition, in addition to mildly acidic pH. Our interpretation is that the PAS domain serves as an “intermediate redox switch” residing between the periplasmic sensor domain and the cytoplasmic catalytic core. This switch is turned on under aerobic conditions via oxidized UQ and gets switched off under anaerobic conditions via reduced form of UQ. The PAS domain integrates two signals: mildly acidic pH and oxidative conditions. The two naphtoquinones, DMK and MK, are presumed to also activate EvgS, but only when EvgS is overexpressed (Figures 8E, 9A). This suggests that oxidized UQ have higher affinity against EvgS than oxidized DMK and MK. On the other hand, reduced DMK and MK are also presumed to inactivate EvgS (Figure 9A), but not the EvgS variants (Figures 9B–D), suggesting that the reduced form of UQ also have higher affinity against EvgS than DMK and MK.

Previous *in vitro* studies on EvgS and BvgS (Bock and Gross, 2002) have shown that the purified soluble forms of the cytoplasmic regions of EvgS and BvgS are inhibited by oxidized UQ-0, with half-maximal inhibition occurring at 4 μM (EvgS) and 11 μM (BvgS). Since these results suggested direct binding of UQ to EvgS, we searched for quinone-binding motifs [aliphatic-(X)_3_-H-(X)_2__/__3_-(L/T/S)] (Fisher and Rich, 2000) within the EvgS PAS domain. As shown in Figure 4A, two putative quinone-binding sites were found, with the central histidines located at 607 and 636, although both sites had mismatches in the number of X residues after the aliphatic residue. Most of the ligands of PAS domains bind to the spatially conserved cleft formed by the inner surface of the β-sheet and the helices Eα and Fα (Möglich et al., 2009). Interestingly, His636 is in helix Fα, suggesting that the latter is a stronger candidate for UQ binding. Alignment of EvgS PAS domains of different *E. coli* strains and *Shigella* species revealed that they are almost identical, and the two putative quinone-binding sites are conserved (Supplementary Figure 10). As for the PAS domain of BvgS, only one putative quinone-binding site in helix Fα with a central His643 has been found (Bock and Gross, 2002). Whether UQ binds to these putative sites in the EvgS PAS domain will be investigated in our future studies.

The EvgS PAS domain has been considered essential for signal transmission, since many different mutations in the PAS domain led to constitutive activation of the protein (Kato et al., 2000; Johnson et al., 2014). The suggested model was that the non-active state of EvgS is a tight inactive dimer, which upon signal perception, changes to a weak active dimer. The PAS domain mutants are thought to weaken the EvgS dimer and cause EvgS activation (Johnson et al., 2014). The third EvgS state is explained as a weaker inactive dimer. This state has been observed in cytoplasmic EvgS mutants, which lack the periplasmic and transmembrane domains and have mutations in the PAS domain (Sen et al., 2017). We have also confirmed that the cytoplasmic EvgS is weakly active and is no longer under redox control (Figure 7). Sen et al. found that adding mutations, which constitutively activate the full-length EvgS, to the PAS domain of the cytoplasmic EvgS caused inactivation (Sen et al., 2017). We assume that this weaker inactive dimer explains the previous *in vitro* results of oxidized UQ inhibiting EvgS (Bock and Gross, 2002). The cytoplasmic EvgS, without the periplasmic and transmembrane domains, forms a weak active dimer. Moreover, the cytoplasmic EvgS_57__9–1197_ used in their study had a truncated N-terminal helix in the PAS domain. The N-terminal helices form a coiled coil that interacts with the β-sheet of the other protomer; truncation of this N-terminal helix weakens the dimer. When oxidized UQ is added to EvgS_57__9–1197_, which is presumed to activate the full-length EvgS in the cell, may further weaken the dimer, resulting in the formation of a weaker inactive dimer.

The present model of EvgS is as follows. Under aerobic conditions, oxidized UQ activates EvgS. MK and DMK can also activate EvgS, but with a lower affinity than UQ. A change to anaerobic conditions inactivates EvgS by reduced UQ. C671 and C683 are presumed to be involved in the redox control. It is of interest that EvgS/EvgA induces the expression of a cytochrome *bd*-II ubiquinol oxidase (encoded by *appCB*) via the YdeO transcription factor (Yamanaka et al., 2014). Cytochrome *bd* is embedded in the prokaryotic cytoplasmic membrane, and produces H_2_O and oxidized UQ upon O_2_ oxidization using UQH_2_ (ubiquinol) as the electron donor (Borisov et al., 2011). The presence of this enzyme and O_2_ increases the ratio of oxidized UQ to UQH_2_. Activation of EvgS/EvgA induces the expression of cytochrome *bd*-II ubiquinol oxidase, and may contribute to the supply of oxidized UQ to maintain the ON state of the EvgS redox switch. Furthermore, Sommer et al. (2013) have reported that EvgS tended to form clusters at the membrane, and that the cytoplasmic PAS domain was required for the clustering.

Recently, another signal has been found for EvgS. Indole, a metabolic product of *E. coli* and other gut bacteria, acts as an inhibitor of EvgS at the micromolar level (Boon et al., 2020). Directly or indirectly, indole affects EvgS activity, and by doing so inhibits induction of the severe acid resistance systems. It has been suggested that indole could enable *E. coli* to regulate gene expression, which fits its location in the gut (Boon et al., 2020). The EvgS PAS domain, acting as an intermediate redox switch, can also adjust *E. coli* to regulate gene expression in accordance with the redox state. Thus, together with the presence of indole, EvgS activity should be repressed in the anaerobic intestine. This is supported by a previous report that showed EvgSA not to have an important role to play under anaerobic conditions (Deng et al., 2013; De Biase and Lund, 2015).

Then, where does EvgS/EvgA function in the environment? There is one report of EvgS/EvgA activation in *Shigella flexneri* (Pasqua et al., 2019). Inside macrophages, *S. flexneri* highly induces the EmrKY efflux pump, which is dependent on EvgS/EvgA. This implies that EvgS is activated in macrophages. Similar to *E. coli*, EvgS in *S. flexneri* is also activated under mildly acidic conditions, which meets the intracellular pH conditions of macrophages. Since the EvgS PAS domain is almost identical between *E. coli* and *S. flexneri* (Supplementary Figure 10), we presume that some kind of oxidative stress that *S. flexneri* faces inside the macrophage turns on the PAS domain. Moreover, a recent report has shown that EvgS/EvgA activation protected *E. coli* from killing by gallium nitrate. This was possibly due to the upregulation of genes encoding enzymes involved in ROS detoxification and in the glyoxylate shunt of the TCA cycle (Zeng et al., 2021). This may also occur in *S. flexneri* and help their survival inside the macrophage. How oxidation of the EvgS PAS domain occurs inside the macrophages is another question that remains to be solved.

## Data Availability Statement

Publicly available datasets were analyzed in this study. This data can be found here: [https://www.biocyc.org/].

## Author Contributions

SI and YE performed the experiments. TO analyzed the structural model of the PAS domain. RU and YE conceived of and supervised the work and wrote the manuscript. All authors contributed to the article and approved the submitted version.

## Conflict of Interest

The authors declare that the research was conducted in the absence of any commercial or financial relationships that could be construed as a potential conflict of interest.

## Footnotes

1 https://swissmodel.expasy.org
