# Supplementary material for: Acid-Sensing Histidine Kinase With a Redox Switch
Source: Front Microbiol. 2021 May 20;12:652546. doi: 10.3389/fmicb.2021.652546 (PMC8174306; doi:10.3389/fmicb.2021.652546)
Supplement: Supplementary file 1 [file Data_Sheet_1.pdf]

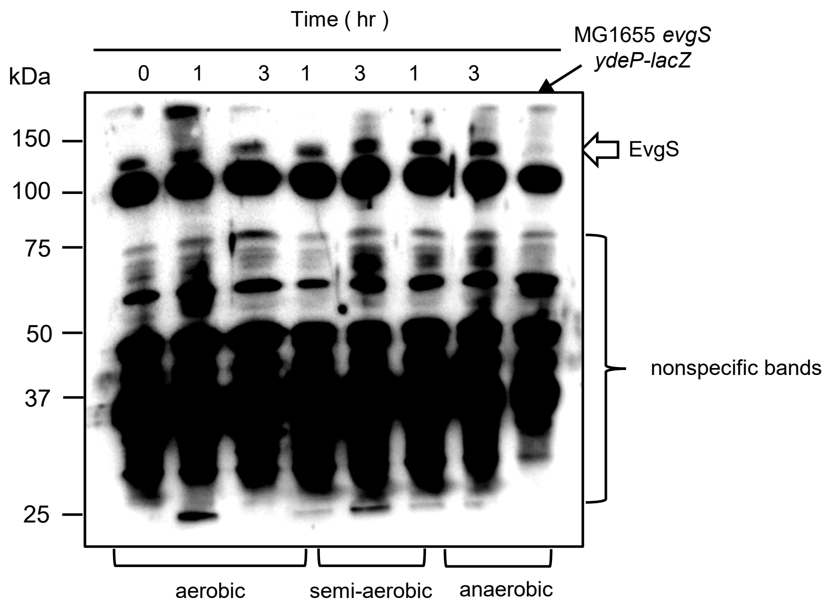

Supplementary FIGURE 1. Full gel version of the immunoblotting results shown in **FIGURE 1B**. The 50 kDa nonspecific bands serve as the loading control.

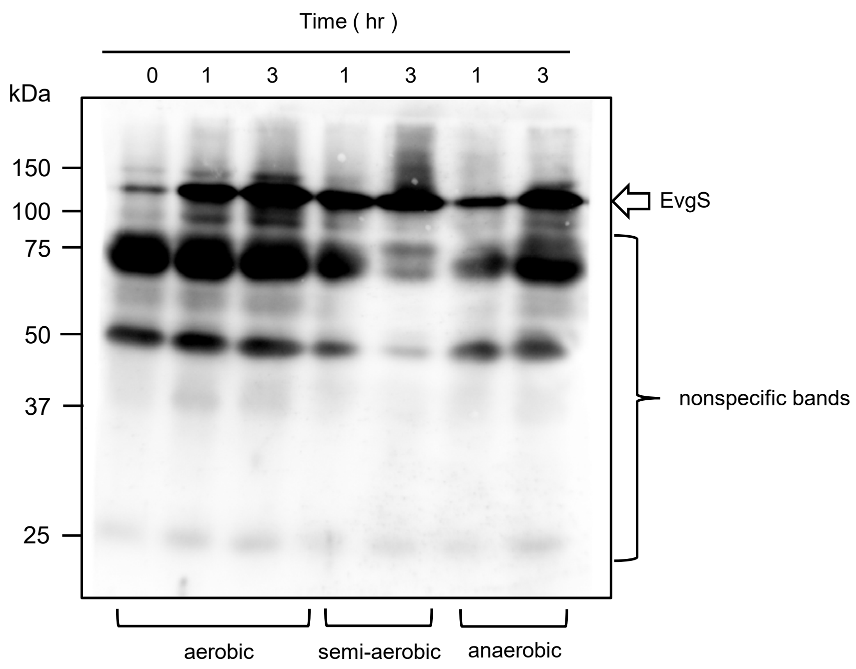

Supplementary FIGURE 2. Full gel version of the immunoblotting results shown in **FIGURE 2B**. The 25 kDa nonspecific bands serve as the loading control.

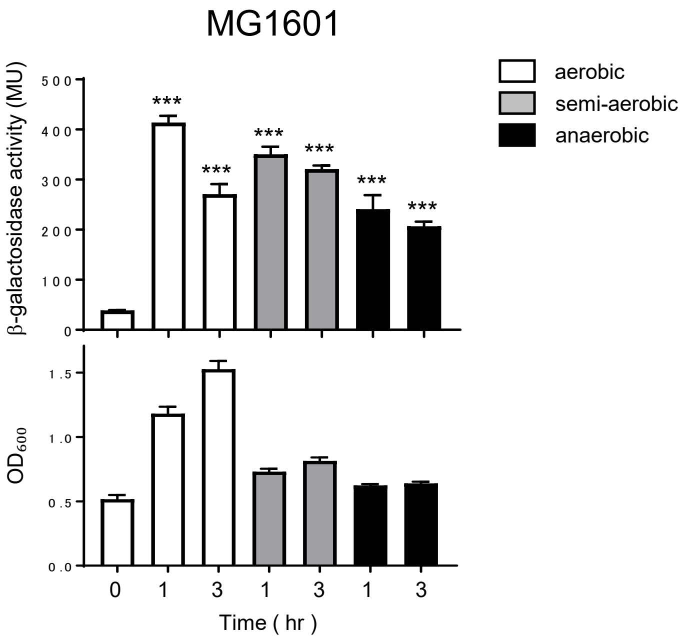

Supplementary FIGURE 3. PhoQ/PhoP system can be activated under anaerobic condition. Activity of the *mgtA* promoter activity is shown in different culturing conditions (upper panel). MG1601 was first grown in PhoQ-inactivating medium (LB + 20 mM $MgSO_4$) until exponential phase, cells collected by centrifugation and resuspended in PhoQ-activation medium (LB with no addition of $MgSO_4$), and grown under aerobic (white bars), semi-aerobic (gray bars), or anaerobic condition (black bars) at 37ºC. Cell growth indicated by optical density at 600 nm is shown in the lower panel. Data represent the average of three biologically independent replicates. Error bars indicate the standard deviation, and statistical analyses of each redox condition group were performed using Dunnett's multiple comparison test with the time 0 sample as the control. ***, $p < 0.001$.

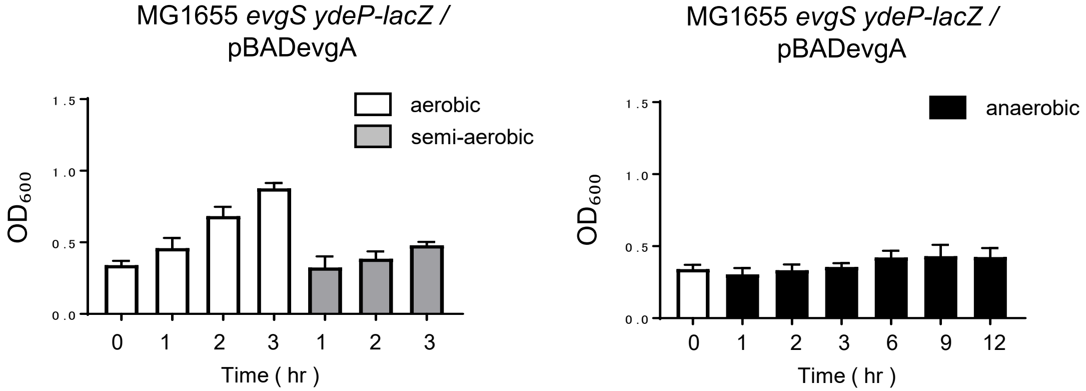

Supplementary FIGURE 4. Opitical density of the cell cultures subjected to reporter assays in **FIGURE 3A**.

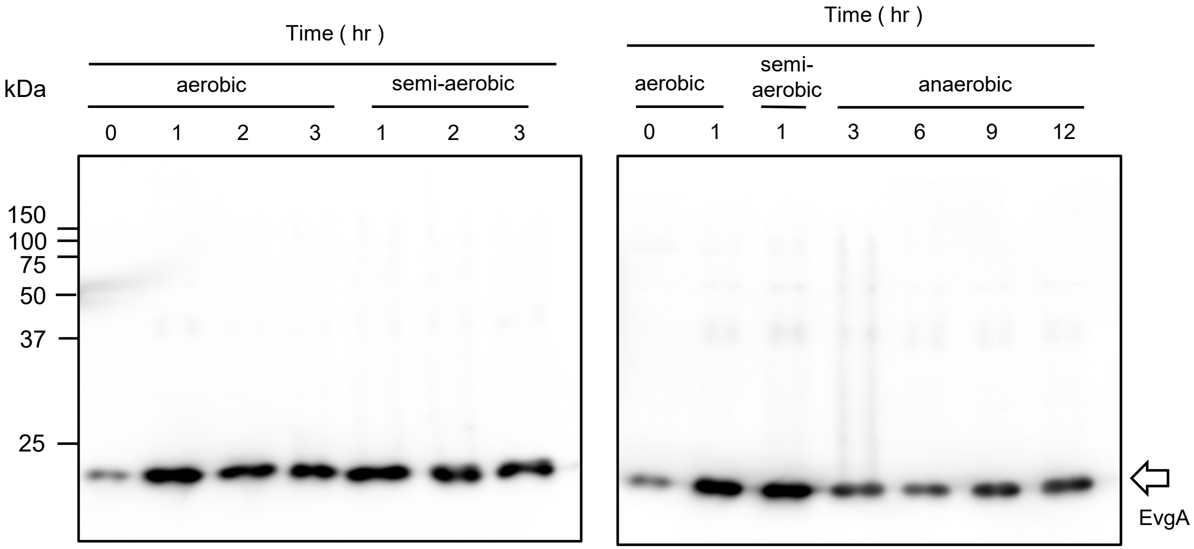

Supplementary FIGURE 5. Full gel version of the immunoblotting results shown in **FIGURE 3B**.

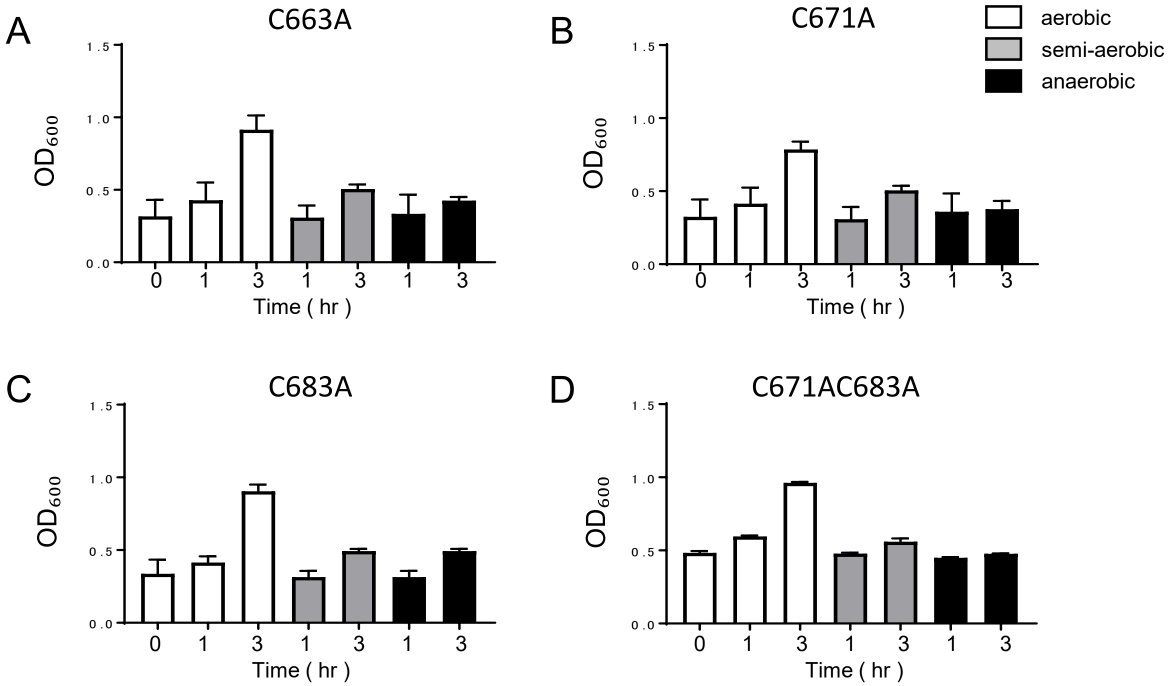

Supplementary FIGURE 6. Opitical density of the cell cultures subjected to reporter assays in **FIGURE 5**.

Inada et al

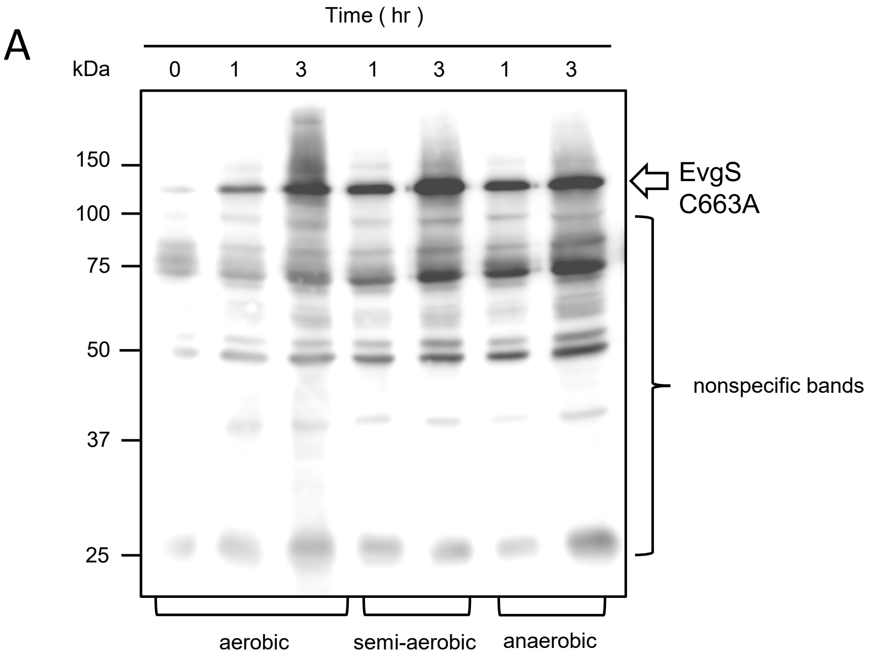

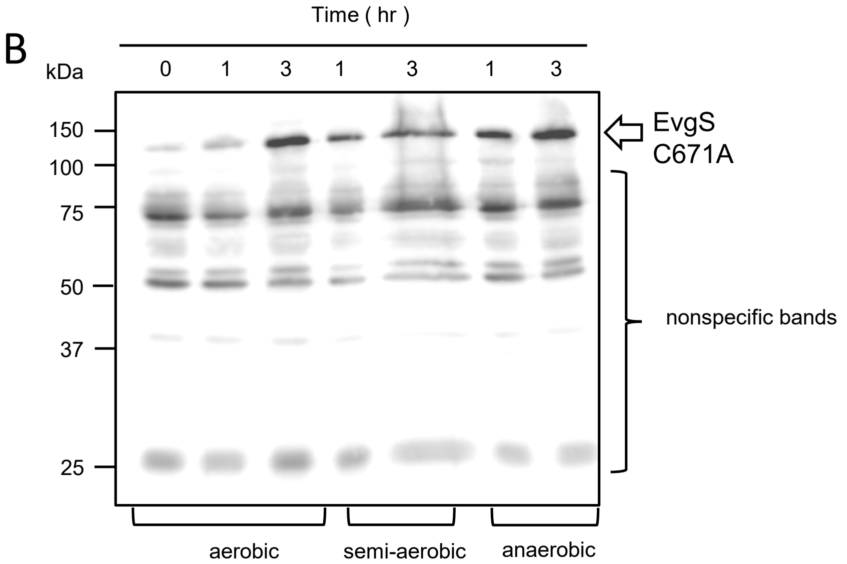

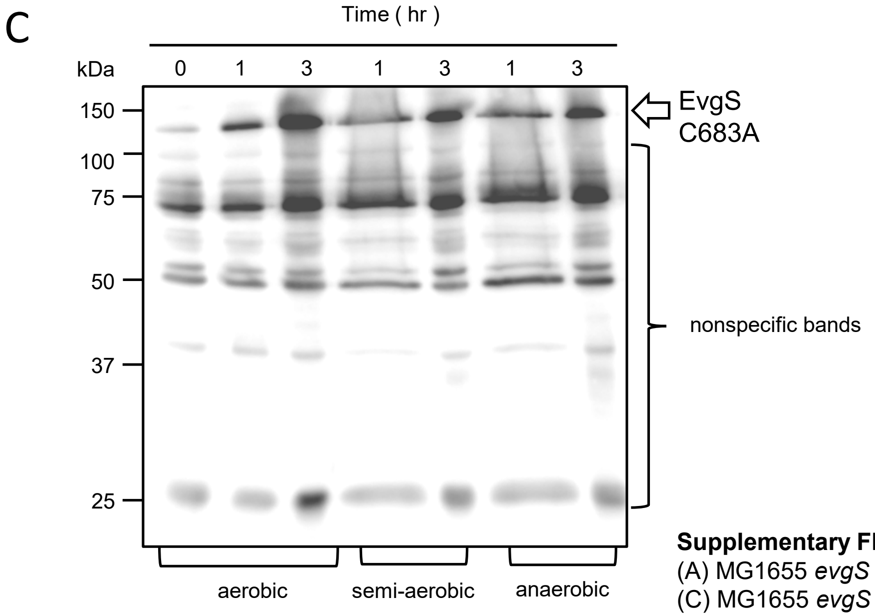

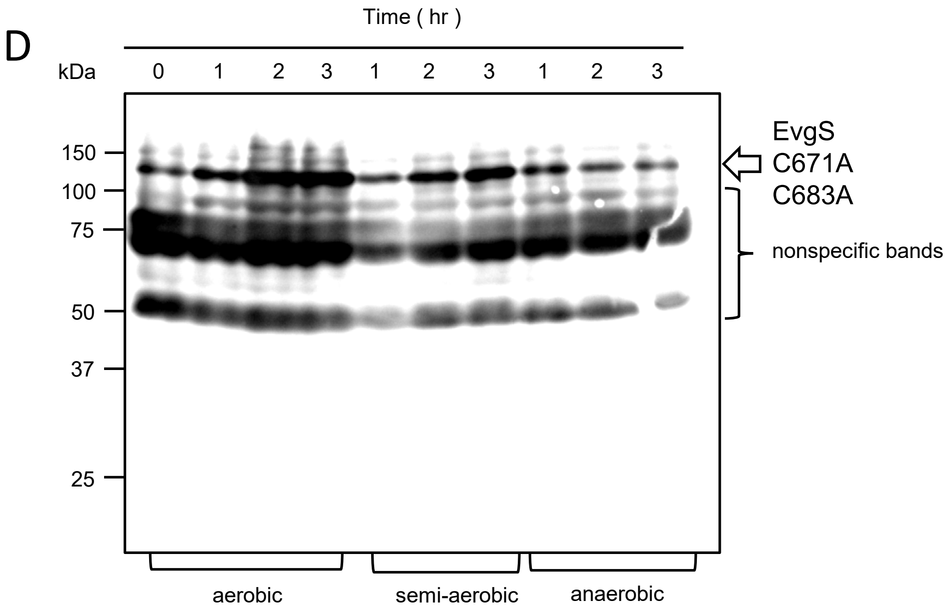

Supplementary FIGURE 7. Expression of EvgS variants.
(A) MG1655 *evgS ydeP-lacZ* / pBADevgS C663A*;* (B) MG1655 *evgS ydeP-lacZ* / pBADevgS C671A*;* (C) MG1655 *evgS ydeP-lacZ* / pBADevgS C683A*,* (D) MG1655 *evgS ydeP-lacZ* / pBADevgS C671A C683A. Immunoblotting analyses using anti-EvgS antiserum for EvgS detection are shown. Samples are from the same culture as those subjected to reporter assays in **FIGURE 5**. The 50 kDa nonspecific bands serve as the loading control.

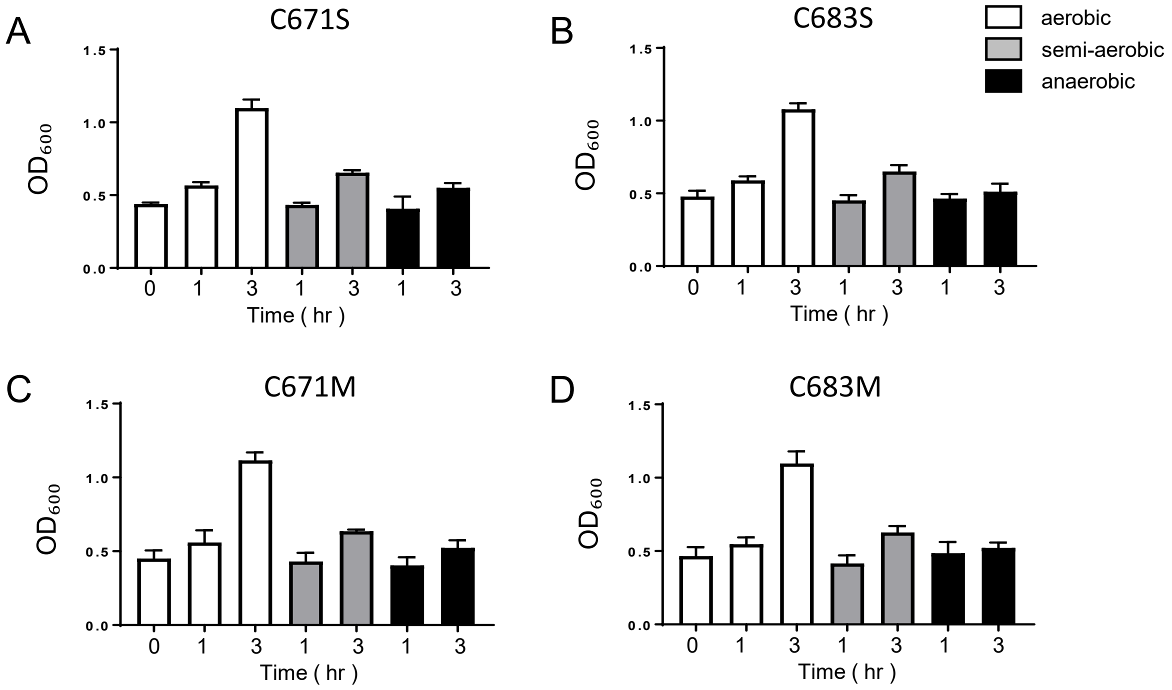

Supplementary FIGURE 8. Opitical density of the cell cultures subjected to reporter assays in **FIGURE 6**.

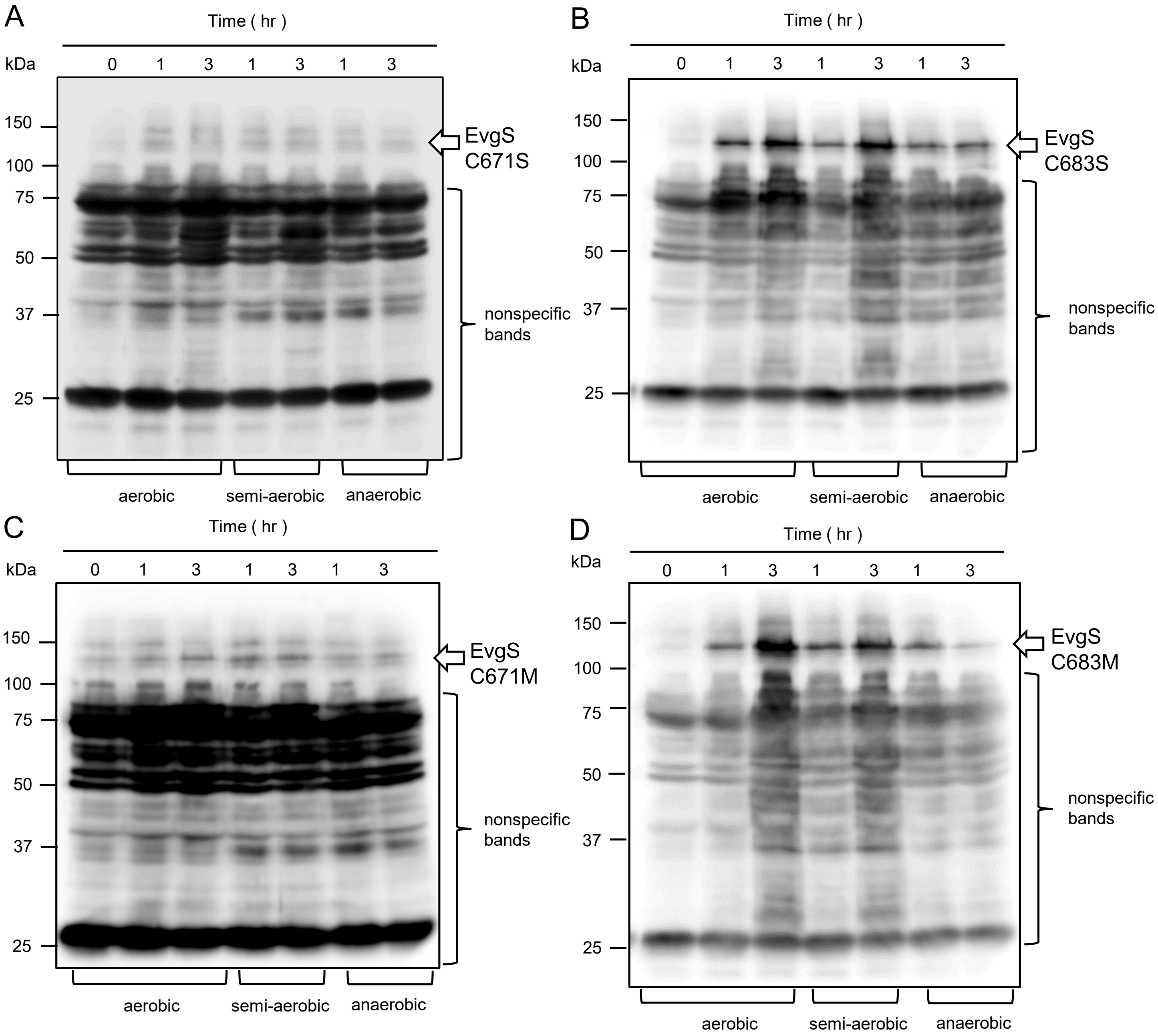

Supplementary FIGURE 9. EvgS expression in EvgS variants. (A) MG1655 *evgS ydeP-lacZ*/ pBADevgS C671S; (B) MG1655 *evgS ydeP-lacZ*/ pBADevgS C683S; (C) MG1655 *evgS ydeP-lacZ*/ pBADevgS C671M; (D) MG1655 *evgS ydeP-lacZ*/ pBADevgS C683M. Immunoblotting analyses using anti-EvgS antiserum for EvgS detection are shown. Samples are from the same culture as those subjected to reporter assays in **FIGURE 6.** The 50 kDa nonspecific bands serve as the loading control.
.

```
                                                  558
E.coli E2348/69           TLSVLLVGSSLLWGFYLLRSVRRRKVIQGDLENQISFRKALSDSLPNPTYVVNWQGNVIS
E.coli UTI89              TLSVLLVGSSLLWGFYLLRSVRRRKVIQGDLENQISFRKALSDSLPNPTYVVNWQGNVIS
E.coli MP1                TLSVLLVGSSLLWGFYLLRSVRRRKVIQGDLENQISFRKALSDSLPNPTYVVNWQGNVIS
E.coli CFT073             TLSVLLVGSSLLWGFYLLRSVRRRKVIQGDLENQISFRKALSDSLPNPTYVVNWQGNVIS
E.coli IAI39              TLSVLLVGSSLLWGFYLLRSVRRRKVIQGDLENQISFRKALSDSLPNPTYVVNWQGNVIS
Shigella_dysenteriae      TLSVLLVGSSLLWGFYLLRSVRRREVIQGDLENQISFRKALSDSLPNPTYVVNWQGNVIS
E.coli UMN026             TLSVLLVGSSLLWGFYLLRSVRRRKVIQGDLENQISFRKALSDSLPNPTYVVNWQGNVIS
E.coli EDL933             TLSVLLVGSSLLWGFYLLRSVRRRKVIQGDLENQISFRKALSDSLPNPTYVVNWQGNVIS
E.coli Sakai              TLSVLLVGSSLLWGFYLLRSVRRRKVIQGDLENQISFRKALSDSLPNPTYVVNWQGNVIS
E.coli MG1655             TLSVLLVGSSLLWGFYLLRSVRRRKVIQGDLENQISFRKALSDSLPNPTYVVNWQGNVIS
E.coli HS                 TLSVLLVGSSLLWGFYLLRSVRRRKVIQGDLENQISFRKALSDSLPNPTYVVNWQGNVIS
Shigella_flexneri         TLSVLLVGSSLLWGFYLLRSVRRRKVIQGDLENQISFRKALSDSLPNPTYVVNWQGNVIS
E.coli 11128              TLSVLLVGSSLLWGFYLLRSVRRRKVIQGDLENQISFRKALSDSLPNPTYVVNWQGNVIS
E.coli 12009              TLSVLLVGSSLLWGFYLLRSVRRRKVIQGDLENQISFRKALSDSLPNPTYVVNWQGNVIS
Shigella boydii ATCC 9210 TLSVLLVGSSLLWGFYLLRSVRRRKVIQGDLENQISFRKALSDSLPNPTYVVNWQGNVIS
Shigella_sonnei_Ss046     TLSVLLVGSSLLWGFYLLRSVRRRKVIQGDLENQISFRKALSDSLPNPTYVVNWQGNVIS
                          ************************:***********************************

E.coli E2348/69           HNSAFEHYFTDDYYKNAMLPLENSESPFKDVFSNTHEVTAETKENRTIYTQVFEIDNGIE
E.coli UTI89              HNSAFEHYFTDDYYKNAMLPLENSESPFKDVFSNTHEVTAETKENRTIYTQVFEIDNGIE
E.coli MP1                HNSAFEHYFTDDYYKNAMLPLENSESPFKDVFSNTHEVTAETKENRTIYTQVFEIDNGIE
E.coli CFT073             HNSAFEHYFTADYYKNAMLPLENSESPFKDVFSNTHEVTAETKENRTIYTQVFEIDNGIE
E.coli IAI39              HNSAFEHYFTADYYKSAMLPLENSESPFKDVFSNTHEVTAETKENRTIYTQVFEIDNGIE
Shigella_dysenteriae      HNSAFEHYFTADYYKNEMLPLENSESPFKDVFSNTHEVTAETKENRTIYTQVFEIDNGIE
E.coli UMN026             HNSAFEHYFTADYYKNAMLPLENSESPFKDVFSNTHEVTAETKENRTIYTQVFEIDNGIE
E.coli EDL933             HNSAFEHYFTADYYKNAMLPLENSESPFKDVFSNTHEVTAETKENRTIYTQVFEIDNGIE
E.coli Sakai              HNSAFEHYFTADYYKNAMLPLENSESPFKDVFSNTHEVTAETKENRTIYTQVFEIDNGIE
E.coli MG1655             HNSAFEHYFTADYYKNAMLPLENSDSPFKDVFSNAHEVTAETKENRTIYTQVFEIDNGIE
E.coli HS                 HNSAFEHYFTVDYYKNAMLPLENSDSPFKDVFSNAHEVTAETKENRTIYTQVFEIDNGIE
Shigella_flexneri         HNSAFEHYFTADYYKNAMLPLENSDSPFKDVFSNAHEVTAETKENRTIYTQVFEIDNGIE
E.coli 11128              HNSAFEHYFTADYYKNAMLPLENSDSPFKDVFSNAHEVTAETKENRTIYTQVFEIDNGIE
E.coli 12009              HNSAFEHYFTADYYKNAMLPLENSDSPFKDVFSNAHEVTAETKENRTIYTQVFEIDNGIE
Shigella boydii ATCC 9210 HNSAFEHYFTADYYKNAMLPLENSDSPFKDVFSNAHEVTAETKENRTIYTQVFEIDNGIE
Shigella_sonnei_Ss046     HNSAFEHYFTADYYKNAMLPLENSDSPFKDVFSNAHEVTAETKENRTIYTQVFEIDNGIE
                          ********** ****. *******:*********:*************************

                                                                               717
E.coli E2348/69           KRCINHWHTLCNLPASEHAVYICGWQDITETRDLIHALEVERNKAINATVAKSQFLATMS
E. coli UTI89             KRCINHWHTLCNLPASEHAVYICGWQDITETRDLIHALEVERNKAINATVAKSQFLATMS
E. coli MP1               KRCINHWHTLCNLPASEHAVYICGWQDITETRDLIHALEVERNKAINATVAKSQFLATMS
E. coli CFT073            KRCINHWHTLCNLPASEHAVYICGWQDITETRDLIHALEVERNKAINATVAKSQFLATMS
E. coli IAI39             KRCINHWHTLCNLPASEHAVYICGWQDITETRDLIHALEVERNKAINATVAKSQFLATMS
Shigella_dysenteriae      KRCINHWHTLCNLPASDHAVYICGWQDITETRDLIHALEVERNKAINATVAKSQFLATMS
E. coil UMN026            KRCINHWHTLCNLPASDHAVYICGWQDITETRDLIHALEVERNKAINATVAKSQFLATMS
E. coil EDL933            KRCINHWHTLCNLPASEHAVYICGWQDITETRDLIHALEVERNKAINATVAKSQFLATMS
E. coli Sakai             KRCINHWHTLCNLPASEHAVYICGWQDITETRDLIHALEVERNKAINATVAKSQFLATMS
E. coli MG1655            KRCINHWHTLCNLPASDNAVYICGWQDITETRDLINALEVEKNKAIKATVAKSQFLATMS
E. coil HS                KRCINHWHTLCNLPASDNAVYICGWQDITETRDLINALEVEKNKAIKATVAKSQFLATMS
Shigella_flexneri         KRCINHWHTLCNLPASDNAVYICGWQDITETRDLINALEVEKNKAIKATVAKSQFLATMS
E. coil 11128             KRCINHWHTLCNLPASDNAVYICGWQDITETRDLINALEVEKNKAIKATVAKSQFLATMS
E.cColi 12009             KRCINHWHTLCNLPASDNAVYICGWQDITETRDLINALEVEKNKAIKATVAKSQFLATMS
Shigella boydii ATCC 9210 KRCINHWHTLCNLPASDHAVYICGWQDITETRDLIHALEVERNKAINATVAKSQFLATMS
Shigella_sonnei_Ss046     KRCINHWHTLCNLPASDHAVYICGWQDITETRDLIHALEVERNKAINATVAKSQFLATMS
                          ****************::****************:******:****:*************
```

Supplementary FIGURE 10. Alignment of Evgs PAS domains (blue, 558 – 717) of *E. coli* strains from different groups and *Shigella* species. C671 and C683 are boxed in red, and putative quinone binding sites are boxed in green. Sequences were obtained from BioCyc (Karp PD et al., Briefings in Bioinformatics, 2019, **20**, 1085-1093), and alignment made by CLUSTALW.

Inada et al

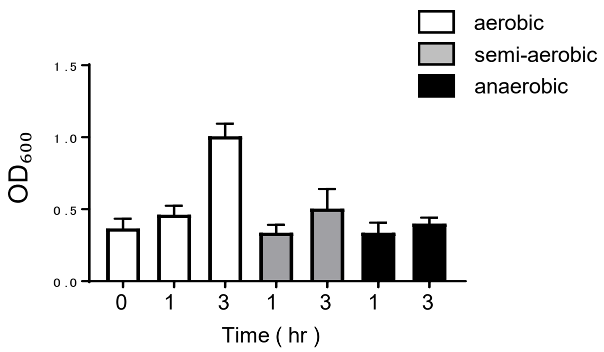

Supplementary FIGURE 11. Opitical density of the cell cultures subjected to reporter assays in **FIGURE 7**.

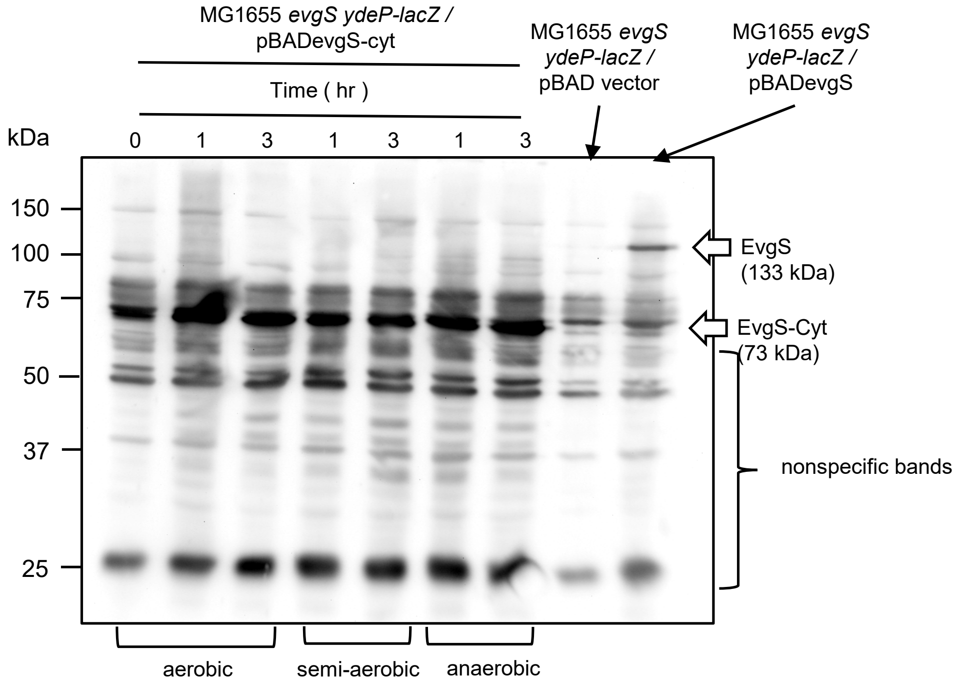

Supplementary FIGURE 12. EvgS-Cyt expression in MG1655 *evgS ydeP-lacZ /* pBAD EvgS-Cyt. Immunoblotting analysis using anti-EvgS antiserum for EvgS detection is shown. Samples are from the same culture as those subjected to reporter assays in **FIGURE 7**. Two lanes at the right show samples from an *evgS* deleted strain and EvgS expressing strain (**FIGURE 2**) for the indication of background bands and full length EvgS band. Note that the EvgS-Cyt band overlaps a non-specific band shown in the MG1655 *evgS ydeP-lacZ* / pBAD vector lane. The 50 kDa nonspecific bands serve as the loading control.

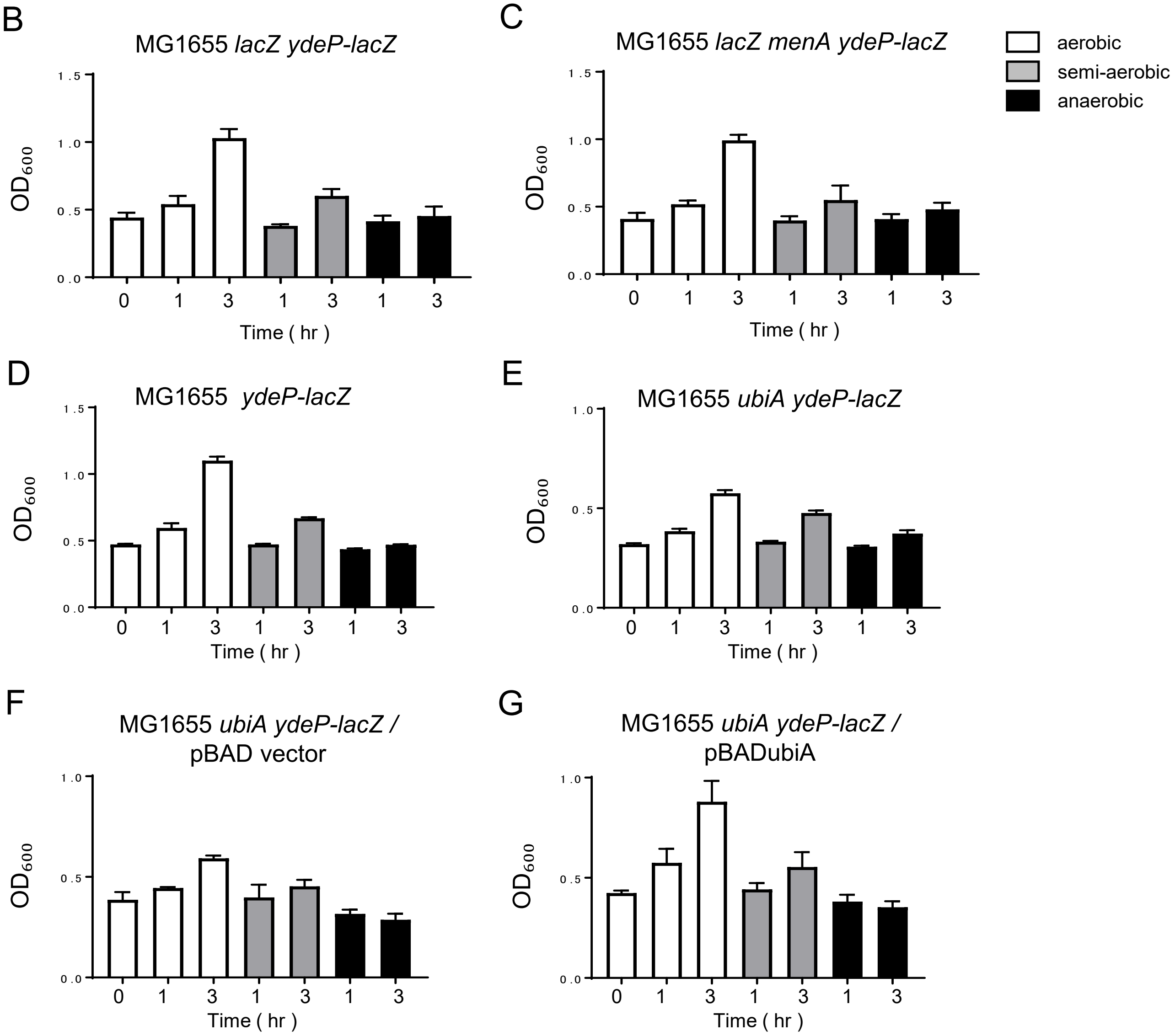

Supplementary FIGURE 13. Opitical density of the cell cultures subjected to reporter assays in **FIGURE 8**.

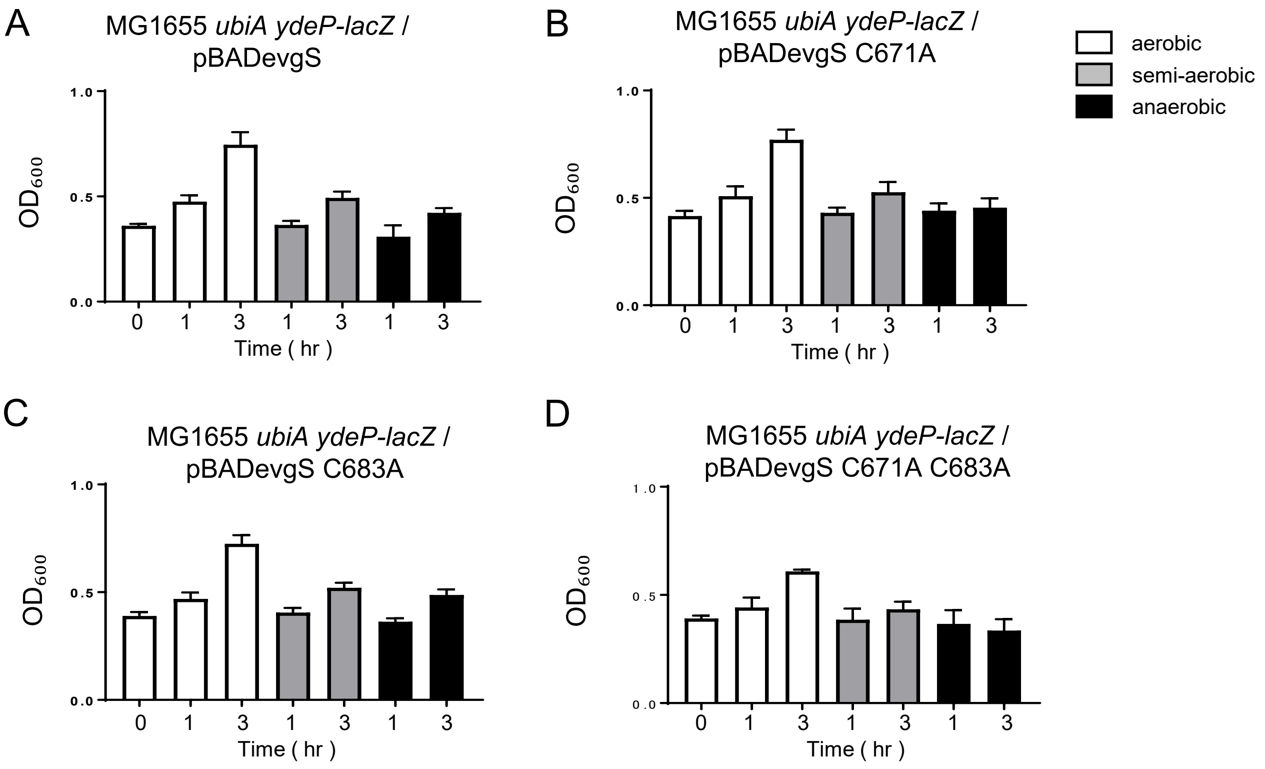

Supplementary FIGURE 14. Opitical density of the cell cultures subjected to reporter assays in **FIGURE 9**.

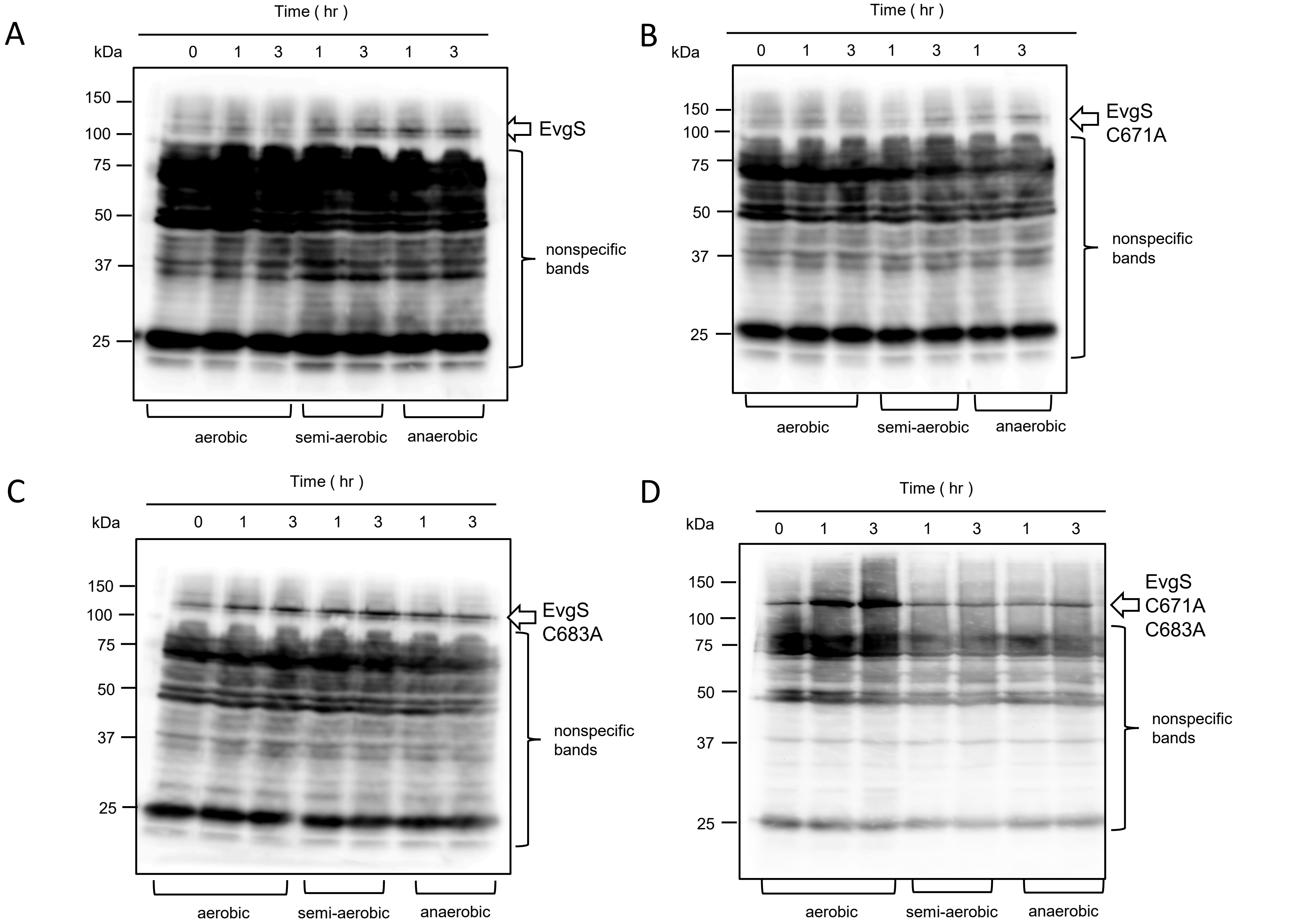

Supplementary FIGURE 15. Expression of EvgS and its variants in MG1655 *ubiA*. (A) MG1655 *ubiA ydeP-lacZ* / pBADevgS*;* (B) MG1655 *ubiA ydeP-lacZ* / pBADevgS C671A*;* (C) MG1655 *ubiA ydeP-lacZ* / pBADevgS C683A*;* (D) MG1655 *ubiA ydeP-lacZ* / pBADevgS C671A C683A. Immunoblotting analyses using anti-EvgS antiserum for EvgS detection are shown. Samples are from the same culture as those subjected to reporter assays in **FIGURE 9**. The 50 kDa nonspecific bands serve as the loading control.
